# Chlorhexidine digluconate mouthwash alters the oral microbial composition and affects the prevalence of antimicrobial resistance genes

**DOI:** 10.3389/fmicb.2024.1429692

**Published:** 2024-06-25

**Authors:** Sibylle Bartsch, Eva Kohnert, Clemens Kreutz, Johan P. Woelber, Annette Anderson, Ann-Sophie Burkhardt, Elmar Hellwig, Wolfgang Buchalla, Karl-Anton Hiller, Petra Ratka-Krueger, Fabian Cieplik, Ali Al-Ahmad

**Affiliations:** ^1^Center for Dental Medicine, Department of Operative Dentistry and Periodontology, Faculty of Medicine and Medical Center, University of Freiburg, Freiburg im Breisgau, Germany; ^2^Institute of Medical Biometry and Statistics, Faculty of Medicine and Medical Center, University of Freiburg, Freiburg im Breisgau, Germany; ^3^Policlinic of Operative Dentistry, Periodontology, and Pediatric Dentistry, Medical Faculty Carl Gustav Carus, Technische Universität Dresden, Dresden, Germany; ^4^Department of Conservative Dentistry and Periodontology, University Hospital Regensburg, Regensburg, Germany

**Keywords:** chlorhexidine, cross-resistance, antibiotics, metagenome, streptococci, caries, efflux pump, tetracycline

## Abstract

**Introduction:**

Chlorhexidine (CHX) is a commonly used antiseptic in situations of limited oral hygiene ability such as after periodontal surgery. However, CHX is also considered as a possible factor in the emergence of cross-resistance to antibiotics. The aim of this study was to analyze the changes in the oral microbiota and the prevalence of antimicrobial resistance genes (ARGs) due to CHX treatment.

**Materials and methods:**

We analyzed the oral metagenome of 20 patients who applied a 0.2% CHX mouthwash twice daily for 4 weeks following periodontal surgical procedures. Saliva and supragingival plaque samples were examined before, directly after 4 weeks, and another 4 weeks after discontinuing the CHX treatment.

**Results:**

Alpha-diversity decreased significantly with CHX use. The Bray–Curtis dissimilarity increased in both sample sites and mainly streptococci showed a higher relative abundance after CHX treatment. Although no significant changes of ARGs could be detected, an increase in prevalence was found for genes that encode for tetracycline efflux pumps.

**Conclusion:**

CHX treatment appears to promote a caries-associated bacterial community and the emergence of tetracycline resistance genes. Future research should focus on CHX-related changes in the microbial community and whether the discovered tetracycline resistance genes promote resistance to CHX.

## Introduction

The World Health Organization (WHO) declared antimicrobial resistance (AMR) as one of the top 10 global public health threats (WHO, 2021a). In this context, the ‘Review on Antimicrobial Resistance’, a project commissioned by the British government, predicts that by 2050 there will be more than 10 million deaths per year due to AMR. The predicted global costs were estimated to exceed USD 100 trillion (O'Neill, 2016). Recently, Murray et al. (2022) estimated that 1.27 million deaths in 2019 could be linked to bacterial AMR alone and concluded that there is a need to expand microbiology research and data collection systems to face this public health threat.

The emergence of AMR is a naturally occurring effect as a response to environmental changes (D’Costa et al., 2011). However, the excessive, incorrectly dosed and unnecessary use of antibiotics has led to an increasing prevalence of AMR due to the high selection pressure (O'Neill, 2016). In addition, a number of other factors can contribute to the emergence of AMR in bacteria. For instance, Cieplik et al. (2019) suggested that the use of antiseptics and biocides had often been underestimated as a driving factor for the evolution of resistance to those antiseptics as well as cross-resistance to important antibiotics.

In this study, we focused on the antiseptic chlorhexidine digluconate (CHX), a bisbiguanide, mainly used for catheter impregnation, skin disinfection, and in oral care (Davies et al., 1954; Lim and Kam, 2008; Brookes et al., 2020; Poppolo Deus and Ouanounou, 2022). It has also a high clinical relevance in periodontology in situations of limited oral hygiene ability such as after periodontal surgery (Solderer et al., 2019). The positively charged CHX interacts at low concentration (0.02–0.06%) with the bacterial cell wall, and has bacteriostatic properties. At high concentrations (>0.1%) it leads to cell leakage, coagulates with cytoplasmic components and has bactericidal effects (Hancock, 1984; McDonnell and Russell, 1999; Gilbert and Moore, 2005; Cieplik et al., 2019; Brookes et al., 2020). Efflux pumps appear to be an important mechanism in the observed AMR associated with the use of antiseptics (Levy, 2002; Russell, 2002; Blanco et al., 2016).

The emergence of CHX-resistant *Proteus*, *Providencia*, and *Pseudomonas* species isolated from urinary tract infections has already been discussed since the 1960s (Lubsen et al., 1961; Bentley et al., 1968; Martin, 1969). Since then, there have been frequent discussions about the intensive use of cationic antiseptics such as CHX, the emergence of antibiotic-resistant microorganisms, and the clinical relevance of these findings (Stickler and Thomas, 1980; Levy, 2002; Russell, 2002; Russell, 2003; Meyer and Cookson, 2010; Kampf, 2016; Cieplik et al., 2019).

As reviewed by Cieplik et al. (2019), the development of AMR due to CHX application in dentistry and its effect on oral microorganisms has mainly been studied on clinical isolates to date, and only more recent studies have also considered the effect on clinical oral isolates and microcosm biofilms (Chatzigiannidou et al., 2020; Schwarz et al., 2021; Mao et al., 2022).

In this context, Russell (2003) indicated that special caution applies when studying resistance to biocides and antibiotics in the laboratory and directly extrapolating these results to the clinical setting. Although some recent microbiome studies were conducted to investigate the effect of CHX on the oral microbiota *in vivo* (Al-Kamel et al., 2019; Tribble et al., 2019; Annavajhala et al., 2020; Bescos et al., 2020; do Amaral et al., 2023), none of these studies investigated the effect of CHX on the prevalence of antimicrobial resistance genes (ARGs). Therefore, it is of utmost importance to examine the impact of CHX on the oral microbiota and the prevalence of ARGs under clinical conditions when applied in dental medicine.

In our metagenomics study, we investigated the effect of frequent CHX use over 4 weeks on the microbial community and resistome in saliva and supragingival plaque. In addition, the recovery of the oral microbiota was studied 4 weeks after discontinuation of CHX treatment.

## Materials and methods

### Study design

The study followed the declaration of Helsinki on human experimentation and was carried out in accordance with Good Clinical Practice. Prior to patient recruitment, the study was approved by the local ethics committee of the University of Freiburg (Reference number: EK-FR 345/19) and registered in the German Clinical Trial Register (DRKS00031475). All patients gave their written informed consent.

Patient recruitment took place from March 2021 to April 2021 in the Department for Operative Dentistry and Periodontology at the University Medical Center Freiburg, Germany, among patients coming for surgical periodontal treatment (either regenerative or plastic periodontal surgery), which indicated the use of CHX rinsing for 4 weeks (twice daily with 0.2% CHX) (Solderer et al., 2019). In cases where the willingness to participate was indicated and informed written consent provided, patients were scheduled for plaque sampling prior to their periodontal surgery, and the collected data are shown in Supplementary Table S1. The sampling started by relative drying using cotton rolls to avoid saliva contamination during sample collection. The supragingival plaque was removed with a sterile curette and pooled directly in an Eppendorf tube with reduced transport fluid (RTF); (Syed and Loesche, 1972) containing 25% glucose and stored at −80°C until analysis. In addition, approx. 1 mL of unstimulated saliva was taken and stored at −80°C for microbiological examinations.

Patients received compensation for their participation. The inclusion and exclusion criteria were as follows:

The inclusion criteria were:

- Surgical periodontal treatment (either regenerative or plastic surgery).
- Age ≥ 18 years.
- Indication for regenerative or plastic periodontal surgery with the indication for post-operative CHX rinsing.

The exclusion criteria were:

- Severe general illnesses.
- Intake of drugs influencing the salivation rate.
- Pregnancy or breastfeeding.
- Active systematic periodontal therapy within the last 2 years.
- Use of antibiotics or antifungals within the last 3 months.
- Allergies against CHX or components of the mouth rinse.

### Metagenomic sequencing

DNA was isolated from supragingival plaque and saliva using the DNeasy® Blood & Tissue Kit (Qiagen, Hilden, Germany) as described by Anderson et al. (2020) with lysozyme (20 mg/mL) and mutanolysin (1,500 U/mL; Sigma-Aldrich, Taufkirchen, Germany), and was incubated for 1.5 h at 37°C. As a positive control, we used our own mock community containing the following microbial species in equal proportions as measured using OD_600_. OD values do not necessarily represent same cell numbers and were only used to estimate the cell quantity: *Fusobacterium nucleatum* (ATCC 25586), *Streptococcus mutans* (DSM 6178), *Streptococcus sanguinis* (DSM 20068), *Streptococcus mitis* (ATCC11843), *Veillonella parvula* (DSM 2008), *Parvimonas micra* (ATCC 33270), *Actinomyces odontolyticus* (DSM 19120), *Neisseria flavescens* (DSM 17633), *Tannerella forsythia* (ATCC 43037), and *Porphyromonas gingivalis* (W381). Colony forming units (CFU) were counted after mixing the mock community, to evaluate if the bacteria proportions were still similar. CFUs were as follows: *Fusobacterium nucleatum* (1×10^7^ CFU/ml), *Streptococcus mutans* (4×10^7^ CFU/ml), *Streptococcus sanguinis* (4×10^7^ CFU/ml), *Streptococcus mitis* (4×10^7^ CFU/ml), *Veillonella parvula* (1×10^7^ CFU/ml), *Parvimonas micra* (1×10^7^ CFU/ml), *Actinomyces odontolyticus* (4×10^6^ CFU/ml), *Neisseria flavescens* (5×10^5^ CFU/ml), *Tannerella forsythia* (1×10^7^ CFU/ml), and *Porphyromonas gingivalis* (1×10^7^ CFU/ml).

The shotgun metagenomic sequencing was performed on an Illumina NovaSeq 6000 S4 PE150 XP [Illumina paired end sequencing (2 × 150 bp)] with a depth of 10 million read pairs (20 million reads) at Eurofins Genomics (Konstanz, Germany). The library was prepared by Eurofins and performed according to their standard genomic library preparation method. The data of the metagenomic sequencing are available through the NCBI Sequence Read Archive (SRA) under the BioProject accession number PRJNA949023.^1^

### Bioinformatics

The raw sequencing data was processed within the bioBakery whole metagenome sequencing workflow (McIver et al., 2018) and human contaminant reads and low-quality reads were removed using KneadData with default values. MetaPhlAn3 (Beghini et al., 2021) was applied to conduct taxonomic profiling and estimation of relative abundances. ARGs were identified with ABRicate, with ARG read annotation only occurring with a sequence identity of at least 90% and coverage of at least 80%. The antibiotics to which a particular ARG confers resistance were determined using the NCBI AMRFinderPlus database. Raw reads were assembled into contigs with metaSPAdes 3.15.4 for which ARGs were identified.

All downstream analyses were conducted in R-Studio (R version 4.1.2) (R Core Team, 2021), and the data was imported into a phyloseq object from the phyloseq R-package (McMurdie and Holmes, 2013), whereby the results are presented either at the species, genus, or phylum level. The genus and phylum level data sets were created by agglomerating the data at the corresponding level. For all statistical tests, a significance level of 5% was applied. Furthermore, Benjamini-Hochberg was used for controlling the false discovery rate (FDR), and adjusted *p*-values are denoted as “p.adj.”

### Data analysis

**Figure 1:**
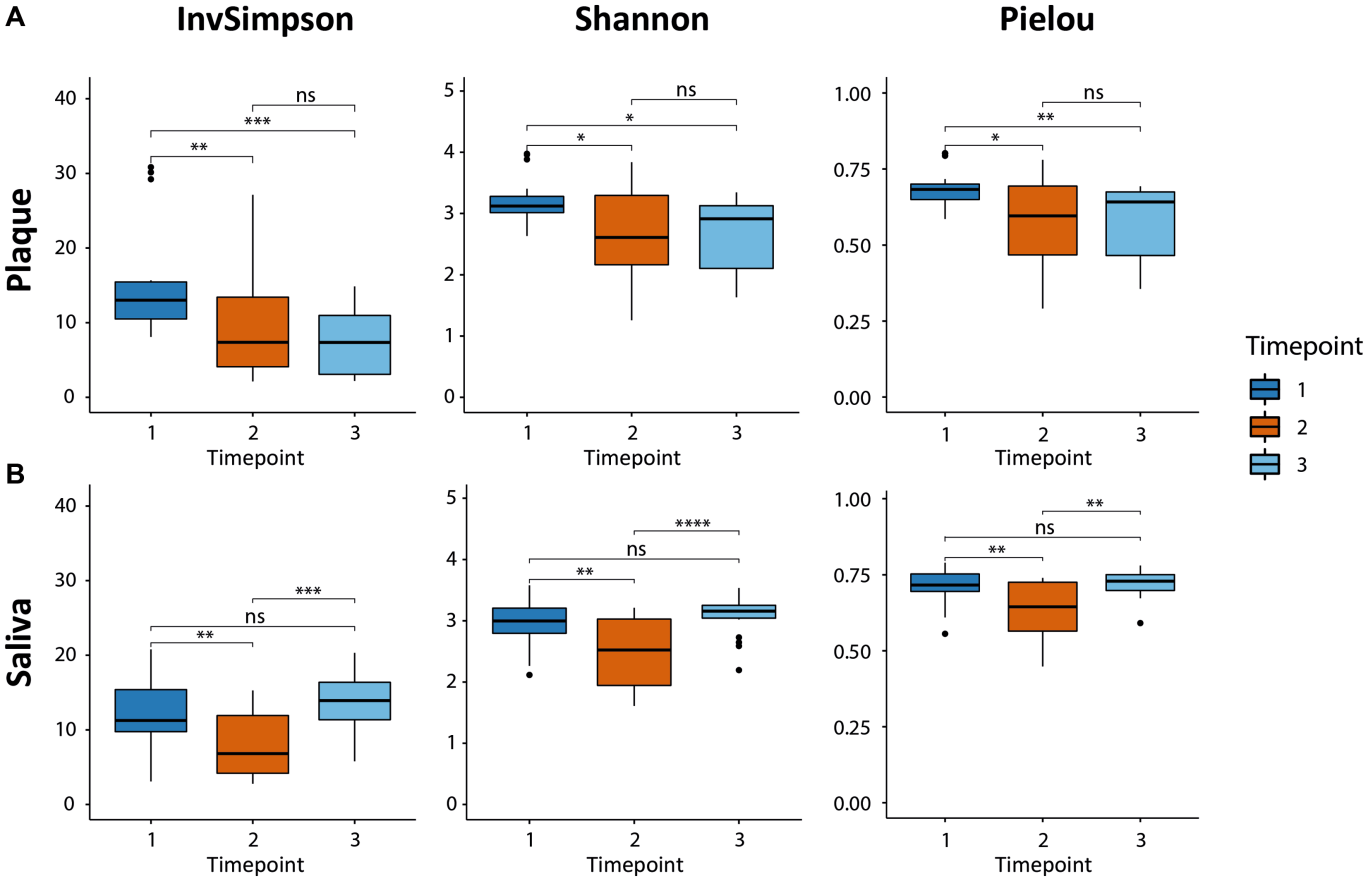
Boxplots of alpha-diversity in both habitats (supragingival plaque and saliva) calculated by three indices, the inverse Simpson diversity index, the Shannon-Weiner diversity index, and the Pielou evenness index. The boxplots show the minimum, 25th percentile, median, 75th percentile, and maximum of the data from bottom to top. **(A)** The microbial diversity in plaque decreased due to CHX use (T2 vs. T1) and could not recover after CHX use was discontinued (T3 vs. T1). **(B)** The diversity in saliva also decreased (T2 vs. T1). The consortium was able to recover and returned to baseline (T3 vs. T1). * p.adj ≤ 0.05; ** p.adj ≤ 0.01; *** p.adj ≤ 0.001; **** p.adj ≤ 0.0001; ns: not significant.

The alpha diversity was analyzed to characterize the bacterial communities within a sample. The inverse Simpson diversity index, the Shannon-Weiner diversity index, and the Pielou’s evenness index were calculated as a measure of alpha-diversity using the vegan package. To test for differences between the timepoints, a paired Wilcoxon signed rank test was applied. Results are presented as boxplots (Figure 1) using the ggplot2 package.

**Figure 2:**
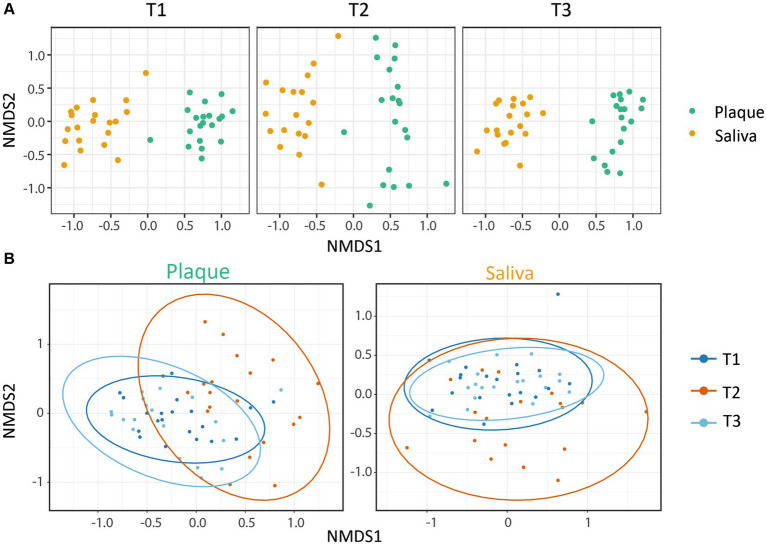
Beta-diversity: NMDS plots based on the Bray-Curtis index. Each point represents a single sample. **(A)** The microbial communities showed a clear clustering dependent on the habitat (saliva or supragingival plaque), whereby both were affected by CHX treatment. **(B)** Within the microbial community of each habitat, CHX use induced a higher dissimilarity. The saliva and plaque communities at T3 were similar to T1, whereas the plaque samples were less alike. Ellipses indicate 95% confidence intervals around cluster centroids. The changes from T1 vs. T2 and T2 vs. T3 were significant (p.adj_Plaque_ ≤ 0.01; p.adj_Saliva_ ≤ 0.05) with an increase in variance for T2.

The microbial similarity between samples was investigated using beta-diversity. The phyloseq package was used to calculate Bray-Curtis distances for dissimilarity measurements and to plot the results using non-metric multidimensional scaling. A pairwise PERMANOVA with 999 permutations based on the adonis function from the vegan package was applied to test for differences in beta-diversity between the timepoints for Figure 2.

**Figure 3:**
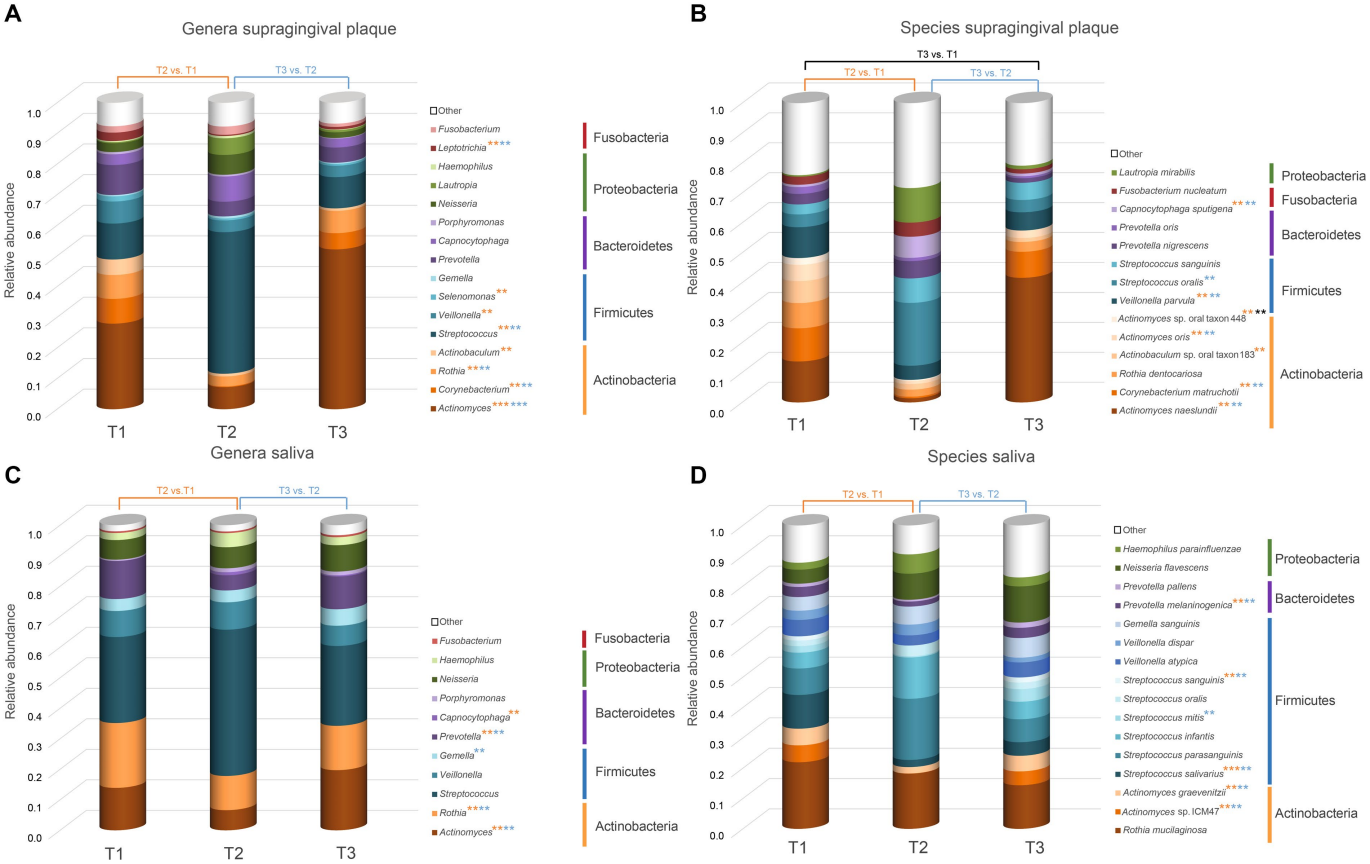
Overview of the microorganisms found in saliva and supragingival plaque communities. Only taxa with a median abundance >1% are represented in the graph. Taxa with a median abundance <1% are summarized as “other.” All genera and species found in this study are listed in the Supplementary Table S2.The values at each timepoint represent the median of 20 patients. **(A)** Genera in supragingival plaque; **(B)** Species in supragingival plaque; **(C)** Genera in saliva; **(D)** Species in saliva. Significant changes are marked with an asterisk (*) in the color corresponding to the change between the timepoints. ** p.adj ≤ 0.01; *** p.adj ≤ 0.001.

Plots representing median abundance across 20 patients of microorganisms with highest overall abundance were generated using excel (Figure 3).

**Figure 4:**
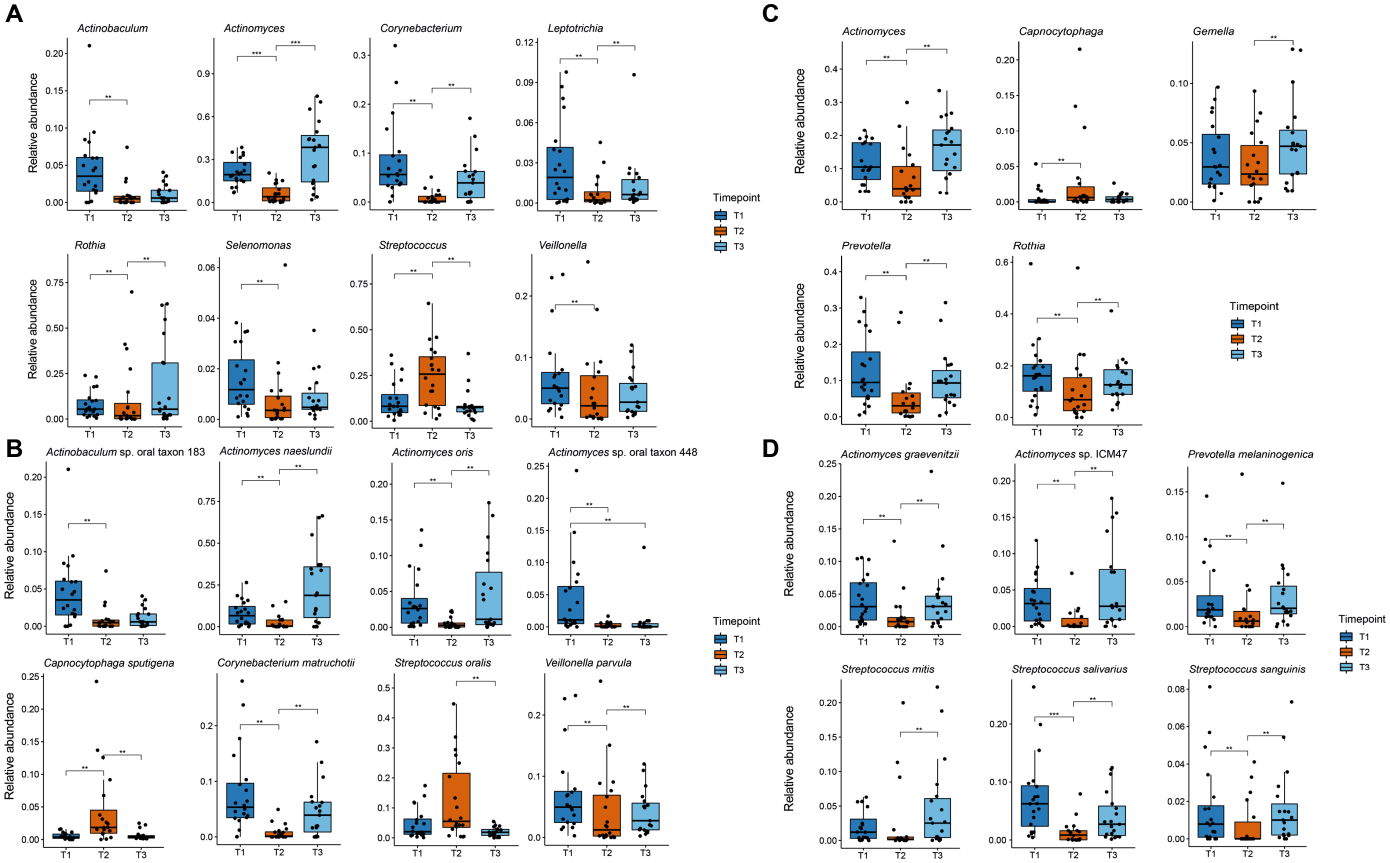
Boxplot of significant changes in the species and genera levels of organisms with a higher relative abundance than 1%. The boxplots show the minimum, 25th percentile, median, 75th percentile, and maximum of the data for all patients. **(A)** Relative abundance of genera in plaque samples showing significant changes; **(B)** Relative abundance of species in plaque samples showing significant changes; **(C)** Relative abundance of genera in saliva samples showing significant changes; **(D)** Relative abundance of species in saliva samples showing significant changes. ** p.adj ≤ 0.01; *** p.adj ≤ 0.001.

Differentially abundant species and genera were identified using mixed linear models as implemented in the Maaslin2 package with the timepoint as a fixed effect and the patient as a random effect (Mallick et al., 2021). For all significantly changed taxa, boxplots were created with the ggplot2 package (Figure 4).

The resistome was characterized by the prevalence of ARGs while the difference in the prevalence of ARGs between timepoints was analyzed as a logistic regression in R using the glm function from the stats package. Plots representing the prevalence of ARGs were generated with the ggplot2 package.

## Results

### Participants

Twenty-one patients were recruited, whereby one participant was a dropout due to antibiotic treatment prior to the study start. Supplementary Table S1 provides an overview of the patients’ characteristics and procedures involved in this study. In general, seven male and 23 female participants aged between 18 and 79 years were included. Two of the patients were smokers. The mean salivary flow rate was 1.75 mL/min (range 1.0–3.6 mL/min), mean salivary pH 7.3 (range 6.5–7.5) and mean salivary buffer capacity was pH 6 (range 5–7).

### Microbial diversity

In our study, we analyzed samples from 20 patients with periodontal diseases before CHX usage (Timepoint 1 – T1), after 4 weeks of daily CHX (0.2%) use (Timepoint 2 – T2), and 4 weeks after CHX use was discontinued (Timepoint 3 – T3). Samples were taken from the supragingival plaque and saliva of the different patients and we first characterized the diversity and (dis)similarities of the microbial communities from the two habitats at the three timepoints.

The alpha-diversity describes the species richness and evenness in the corresponding habitat and was calculated using three different indices (Figure 1), all of which yielded similar results concerning the behavior of the microbial communities for the respective timepoints and samples. The microbial community in the supragingival plaque displayed a significant decrease in diversity after CHX use (T2) in comparison to T1 (Figure 1A). After the discontinuation of CHX use (T3), alpha-diversity increased again, but not significantly and did not reach the baseline value from T1. The plaque community was thus evidently not able to recover to its initial diversity 4 weeks after the CHX use was discontinued. The microbial community in the saliva samples also showed a significant drop in diversity after the CHX treatment (T1 vs. T2) but, in contrast to the community in plaque, it was able to fully recover when the CHX use was discontinued (T3) (Figure 1B).

To gain information about the differences and similarities of the microbiota between the different specimens and patients, we calculated the beta-diversity based on the Bray-Curtis distance. Figure 2 shows the non-metric multidimensional scaling (NMDS) plots that were constructed to identify the clustering patterns of the samples. The results display a clear clustering within the samples of saliva and plaque respectively, demonstrating the dependence of the microbial community on the niche. It can also be observed that CHX use led to significantly higher dissimilarities between the communities, although these still cluster according to their habitats. After discontinuation of CHX use, the community compositions were able to slightly equalize to T1 (Figure 2A). Figure 2B also illustrates the variance in the composition of the bacterial community in each habitat before, during and after CHX use. Again, the dissimilarity of the bacterial community increased at T2, during CHX treatment, which can be explained by a shift in the composition of the microbial community. After discontinuation of CHX (T3), the bacterial community showed the ability to recover from the antimicrobial agent and returned to a composition comparable to that observed at T1. Changes between T1 and T2 as well as between T2 and T3 were significant (Figure 2B). In addition, as also seen in the alpha-diversity plots, the saliva community was able to recover almost to the initial stage, whereas this was less pronounced for the plaque community (Figure 2B).

### Microbial community composition

Overall, we found 74 different genera and 247 species in the supragingival plaque community and 66 different genera and 223 species in the saliva community, respectively (Supplementary Table S2). In the mock community, which we used as a positive control for the DNA isolation and shotgun sequencing procedure, all taxa could be detected and the relative abundance of each species within the mock community was similar at each of the three timepoints (Supplementary Figure S1). Differential abundance of the species themselves seem rather be due to lysis efficiency than to CFU.

Figure 3 shows the relative abundance of all genera and species with a median relative abundance of at least 1% in the saliva and plaque samples, respectively. In supragingival plaque, the microbial community composition at T1 was mainly comprised of Actinobacteria, followed by Firmicutes and Bacteroidetes. The main genera were *Actinomyces* (19.5%), *Streptococcus* (8.2%), *Prevotella* (6.8%), *Corynebacterium* (5.6%), *Rothia* (5.4%), and *Veillonella* (5.0%). After the patients used CHX twice daily for four weeks (T2), the biofilm communities changed to a significant predominance of the genus *Streptococcus* (25.8%) (Figures 3A, 4A). The genera *Capnocytophaga* (4.6%), *Neisseria* (3.6%), and *Lautropia* (3.0%) also increased but at low relative levels and these results were not significant (Figure 3A and Supplementary Figure S2A). While the relative abundance of *Actinomyces* dropped significantly, it became even more predominant at T3 when CHX use was discontinued, whereas the number of streptococci (7.5%) decreased significantly. The most abundant species detected at T1 in supragingival plaque were *Actinomyces naeslundii* (6.6%), *Corynebacterium matruchotii* (5.3%), *Veillonella parvula* (5.0%), and *Rothia dentocariosa* (4.0%) and, although their relative abundance dropped significantly during CHX treatment, all species could recover after the treatment was discontinued. Furthermore, *A. naeslundii* (18.8%) showed a much higher relative abundance at T3 than at T1 while *Streptococcus oralis* (5.5%)*, Lautropia mirabilis* (3.0%), and *Capnocytophaga sputigena* (1.9%) displayed a tendency to increase at T2 after CHX treatment and decreased again after the treatment had stopped. However, only the increase of *C. sputigena* at T2 was significant (Figures 3B, 4B and Supplementary Figure S2B).

At T1 in saliva, we mainly found Firmicutes followed by Actinobacteria. Overall, the predominant species were those belonging to the genera *Streptococcus* (21.3%) and *Rothia* (16.0%) in addition to *Actinomyces* (10.5%), *Prevotella* (9.5%), and *Veillonella* (6.5%) (Figure 3C). The dominant species here were *Rothia mucilaginosa* (12.4%), *Streptococcus salivarius* (6.5%), and *Streptococcus parasanguinis* (4.9%). After CHX treatment, the relative abundance of Firmicutes increased even more with streptococci (29.0%) becoming predominant. *S. parasanguinis* (7.5%) and *S. infantis* (5.1%) were the most abundant species (Figure 3D) although these increases at T2 showed no significance (Supplementary Figures S2A,B). After the discontinuation of CHX use, the relative abundance of several species increased again (T3 vs. T2). While streptococci remained dominant (22.7%), *Actinomyces* (17.1%), *Rothia* (12.6%), and *Prevotella* (9.3%) again increased significantly (Figure 4C) which aligns with the recovery of the alpha- and beta-diversity of the community that was observed (Figures 1, 2).

### Prevalence of antimicrobial resistance genes

**Figure 5:**
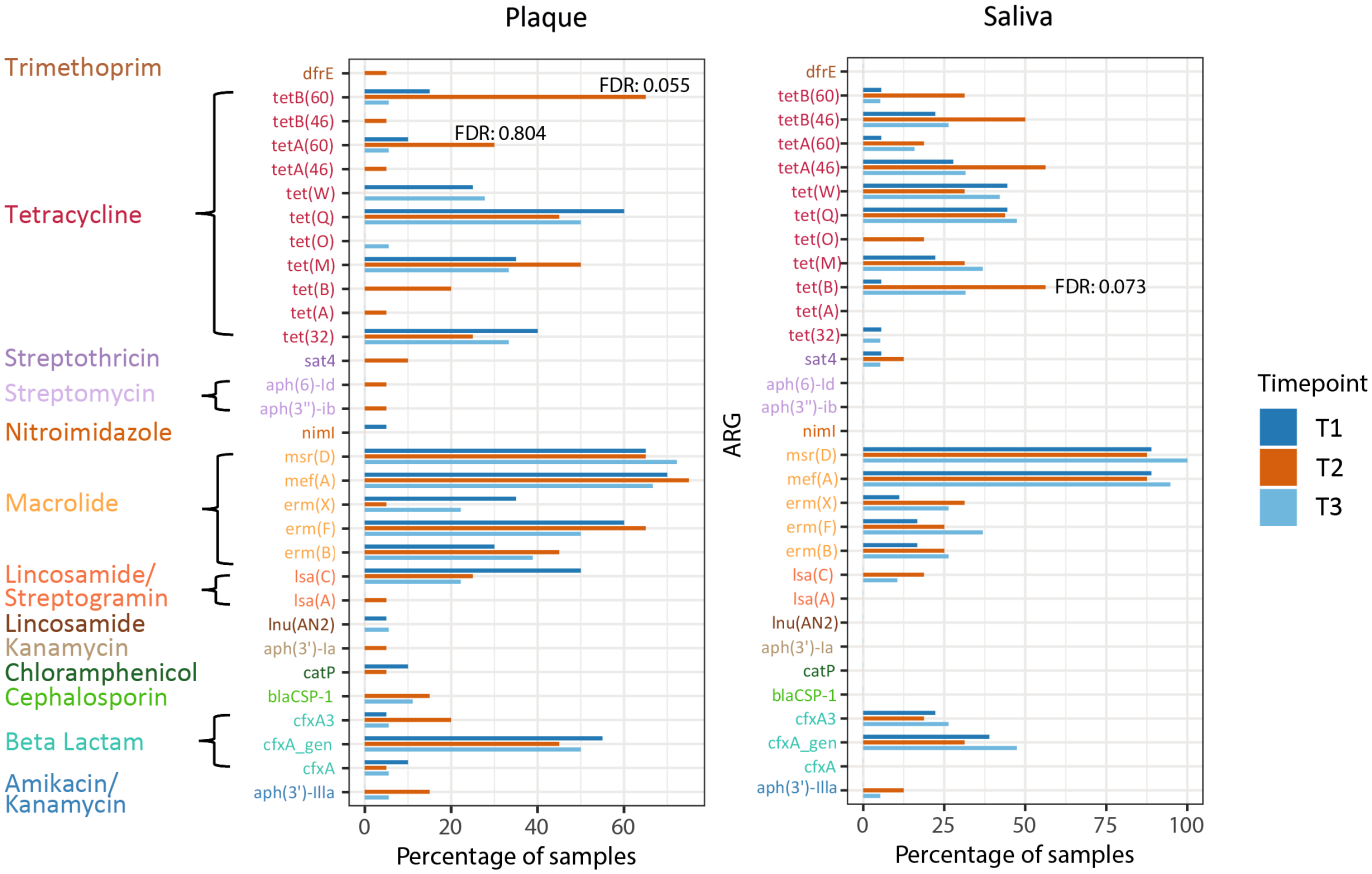
Prevalence of ARGs in plaque and saliva at the different timepoints. The false discovery rate (FDR) (T2 vs. T1) of tetB(60) and tetA (60) in supragingival plaque and of tet(B) in saliva are specified.

In addition to examining the relative abundance of the different microorganisms in the oral habitats, we investigated the prevalence of ARGs in relation to CHX treatment in the patients. The most prevalent ARGs in supragingival plaque across all timepoints were the macrolide resistance genes *mef*(A), *msr*(D), and *erm*(F), the tetracycline resistance gene *tet*(Q), and beta-lactamase *cfx*A. In saliva, ARGs were less prevalent, with *mef*(A) and *msr*(D) showing the highest prevalence with more than 80%. None of these genes displayed a significant change in prevalence during CHX treatment and, overall, no significant enrichments in ARGs were found in any of the habitats at T2. However, some tetracycline ARGs showed a non-significant increase at T2 compared to T1 (Figure 5). In supragingival plaque, there was a tendency for the enrichment of the genes *tetA*(60) and *tetB*(60) after CHX treatment (FDR: 0.055 and 0.804, respectively) (Supplementary Table S3). In saliva, we could also observe a higher prevalence of a tetracycline resistance gene *tet*(B) at T2 vs. T1 (FDR: 0.073) (Supplementary Table S3).

## Discussion

CHX is widely used in dentistry and oral care and can be considered the gold-standard oral antiseptic (Jones, 1997; Cieplik et al., 2019). As a result, the impact of CHX mouthwash use on microbial communities and the development of resistance to this antiseptic, as well as cross-resistance to antibiotics, is an important area of current research (Cieplik et al., 2019). Therefore, our study investigated the changes in the microbial communities and the resistome *in vivo* during 0.2% CHX treatment. This concentration had an initial bactericidal effect, which decreased due to dilution by the saliva and supragingival biofilm.

We observed in our study a change in alpha- and beta-diversity in supragingival plaque and saliva as a result of using a CHX mouthwash twice daily for 4 weeks. While the alpha-diversity decreased during CHX treatment, when CHX was discontinued, it was able to completely recover in saliva after 4 weeks, whereas in supragingival plaque, the microbial community was not able to recover after this time. In a study by Jing et al. (2019), biofilms treated with antibacterial agents such as CHX for 10 min needed 15 weeks to fully return to their pretreatment levels and it is thus reasonable to assume that the plaque community in our study may also regain its diversity after a longer recovery period. Similar results were observed in both habitats for the Bray-Curtis beta-diversity. Here, CHX induced a higher dissimilarity within the observed clusters, which aligned at T3 to similar levels as at T1, with less convergence in plaque again being observed.

Similar results were obtained in other studies on the effects of CHX on microbial communities. Al-Kamel et al. (2019) and Bescos et al. (2020) examined the impact of CHX on *in vivo* microbial communities in subgingival plaque and saliva from healthy subjects, respectively and both also observed a drop in alpha-diversity and higher dissimilarities in beta-diversity. Furthermore, Tribble et al. (2019) reported a decrease in species richness on the tongue while using CHX. However, in contrast, Annavajhala et al. (2020) did not observe any changes in alpha-diversity after CHX treatment in microbial communities cultured from saliva and oral swabs, although alterations in beta-diversity were found.

When examining the community composition in more detail, we observed the typical bacteria phyla and genera belonging to a healthy core microbiome (Zaura et al., 2009; Dewhirst et al., 2010; Wade, 2013; Hall et al., 2017; Joseph and Curtis, 2021). We found an initial dominance of *Actinomyces* spp. in supragingival plaque, with *Actinomyces naeslundii* as the most important plaque former. Other main species in the biofilm were *Corynebacterium matruchotii*, *Veillonella parvula* and *Rothia dentocariosa* – all specialized to dental plaque (Mark Welch et al., 2019). The most abundant taxa at the species-level found in saliva (*Rothia mucilaginosa, Streptococcus salivarius, Streptococcus parasanguinis, Actinomyces graevenitzii,* and *Veillonella atypica*) differ from those in plaque and are also in line with the biogeography of non-plaque specific organisms described by Mark Welch et al. (2019). This habitat specificity is also consistent with the observed clustering in beta-diversity.

We observed an increasing relative abundance of *Streptococcus* spp. during the use of the CHX mouthwash in both habitats. Streptococci are associated with oral health if compared with periodontitis patients (Griffen et al., 2012; Belstrøm et al., 2016). However, the shift to an increased number of streptococci was also observed in the saliva and subgingival plaque of two other metagenomic studies with healthy patients and also in microcosm biofilms, all treated with CHX (Al-Kamel et al., 2019; Bescos et al., 2020; Mao et al., 2022). Reduced susceptibility of several *S. oralis* strains against CHX could also be shown in a study by Früh et al. (2022). Furthermore, Bescos et al. (2020) also found a negative effect of CHX on saliva-buffering capacity. Since oral streptococci are acid-producing and acid-tolerant organisms and also non-mutans streptococci are associated with caries, our findings support the suggestions from Bescos et al. (2020) and Mao et al. (2022) of the development of a more caries-associated bacterial community through CHX use (Sansone et al., 1993; Alam et al., 2000; de Soet et al., 2000; Quivey et al., 2001; Beighton, 2005). Therefore, as also reviewed by Walsh et al. (2015), it seems reasonable to critically rethink the application of CHX in caries prevention.

In both, supragingival plaque and saliva, *mef*(A) and *msr*(D) were the ARGs with the highest prevalence over all timepoints. The gene *mef*(A) belongs to the macrolide efflux (*mef*) family while *msr*(D) belongs to the ATP-binding cassette (ABC) proteins, first described in *Streptococcus pneumoniae* (Clancy et al., 1996; Daly et al., 2004). It is hypothesized that both genes belong to the *mef*(A)–*msr*(D) efflux transport system, leading to macrolide resistance (e.g., azithromycin) (Ambrose et al., 2005; Nunez-Samudio and Chesneau, 2013; Iannelli et al., 2018; Fox et al., 2021). Additionally, *erm*(F) and *tet*(Q) were found in more than 50% of the plaque samples. The gene *erm*(F) confers resistance to macrolides and lincosamides (e.g., clindamycin) while *cfxA* encodes for a beta-lactamase (Fosse et al., 2002; Arredondo et al., 2019a,b). In 2017, clindamycin and the beta lactam antibiotic amoxicillin were among the most prescribed antibiotics in dentistry (Thompson et al., 2022). In the previous year, the European Medicines Agency (EMA) initiated a review of azithromycin-containing medicines due to the emergence of resistance to these drugs in the EU (EMA, 2023).

Our results reveal a high prevalence of these genes independent from CHX treatment. Anderson et al. (2023) also found that these five ARGs were most prevalent in the oral cavity.

In contrast, *tetB*(60) displayed a high tendency to increase in prevalence during CHX use in supragingival plaque. In addition to the resistance to tetracycline, this gene – together with *tetA*(60) – encodes the resistance to tigecycline, which is one of the last-resort antibiotics (Reynolds et al., 2016; WHO, 2021b; Brooks et al., 2022). To date, nothing is known about the prevalence of this ABC transporter in the microbiome of the human oral cavity.

The *tet*(B) gene also encodes for an efflux pump (MFS transporter), found in saliva samples with high prevalence after CHX treatment. This gene confers resistance to tetracycline, doxycycline, and minocycline but not tigecycline and is one of the most commonly carried efflux genes identified in Gram-negative bacteria (Roberts, 2005; Grossman, 2016). Chander et al. (2011). discovered the *tet*(B) gene in plasmid extracts from *Streptococcus suis* isolated from pigs (Chander et al., 2011), which was the first time, a detection of this gene in Gram-positive bacteria was presented. The authors assumed a horizontal gene transfer due to selection pressure because of widespread antibiotic use in pig farming. In 2019, *tet*(B) was first found in *S. oralis* isolated from the human gingival sulci (Arredondo et al., 2019a,b). This is a further indication of the evolution of resistance genes in the food industry and transfer to the human body, which has already been hypothesized in various studies and reviewed by Verraes et al. (2013).

Additionally, Royer et al. (2022) identified a *tet*A efflux gene that led to reduced sensitivity of *Escherichia coli* to CHX and tetracycline. This supports our results concerning the potential role of *tetB*(60) and *tet*(B) during CHX treatment. The acquisition of *tet* genes in streptococci may be a contributing factor to the reduced sensitivity to CHX. However, further experiments are necessary to elucidate this phenomenon. In particular, screening of resistance genes in isolated streptococci would be necessary.

In summary, CHX decreased the microbial diversity and reduced the relative abundance of several bacterial taxa. The increased relative abundance of streptococci forming a possible caries-associated bacterial community and also the possible link to the increased prevalence of *tetB*(60) and *tet*(B) resistance genes after CHX treatment need to be further investigated.

In general, the oral cavity is a reservoir for ARGs, which can spread, through biofilm sloughing and swallowing, from the saliva into the human gut, leading to further spread of these genes. This leads to a reduction in the effectiveness of antibiotics and other antimicrobial drugs, making it more difficult to treat infections. Therefore, it is of utmost importance to take measures to decrease the selection pressure in the oral cavity (by local and systemic treatments) to reduce the occurrence and transmission of ARGs in oral bacteria. This can be achieved by employing antibiotics and antibacterial substances such as chlorhexidine (CHX) in a prudent manner.

The results of our study do not, in themselves, constitute a clinical contraindication to the use of CHX. Nevertheless, it is necessary to consider whether the use of CHX is appropriate in the absence of a clinical indication and whether it should be a component of over-the-counter products. Consequently, further research should be conducted in order to ascertain its clinical relevance more accurately. It is important for dentists to be aware of the potential risks associated with widespread use of antiseptics such as CHX.

## Data availability statement

The datasets presented in this study can be found in online repositories. The names of the repository/repositories and accession number(s) can be found at: https://www.ncbi.nlm.nih.gov/, PRJNA949023.

## Ethics statement

The studies involving humans were approved by Ethics Committee of the University of Freiburg, Freiburg, Germany. The studies were conducted in accordance with the local legislation and institutional requirements. The participants provided their written informed consent to participate in this study.

## Author contributions

SB: Formal analysis, Visualization, Writing – original draft, Writing – review & editing. EK: Data curation, Formal analysis, Visualization, Writing – original draft. CK: Data curation, Formal analysis, Visualization, Writing – review & editing. JW: Conceptualization, Methodology, Writing – review & editing. AA: Data curation, Methodology, Writing – review & editing. A-SB: Methodology, Writing – review & editing. EH: Conceptualization, Writing – review & editing. WB: Conceptualization, Writing – review & editing. K-AH: Conceptualization, Writing – review & editing. PR-K: Conceptualization, Writing – review & editing. FC: Conceptualization, Funding acquisition, Investigation, Writing – review & editing. AA-A: Conceptualization, Funding acquisition, Investigation, Writing – review & editing.

## Footnotes

^1^https://www.ncbi.nlm.nih.gov/sra/PRJNA949023
