# Supplementary material for: Chlorhexidine digluconate mouthwash alters the oral microbial composition and affects the prevalence of antimicrobial resistance genes
Source: Front Microbiol. 2024 Jun 25;15:1429692. doi: 10.3389/fmicb.2024.1429692 (PMC11231401; doi:10.3389/fmicb.2024.1429692)
Supplement: Supplementary file 1 [file Data_Sheet_1.PDF]

Chlorhexidine digluconate mouthwash alters the oral microbial composition and affects the prevalence of antimicrobial resistance genes

Sibylle Bartsch, Eva Kohnert, Clemens Kreutz, Johan P. Woelber, Annette Anderson, Ann-Sophie Burkhardt, Elmar Hellwig, Wolfgang Buchalla, Karl-Anton Hiller, Petra Ratka-Krueger, Fabian Cieplik, Ali Al-Ahmad

Appendix

Supplementary Figures

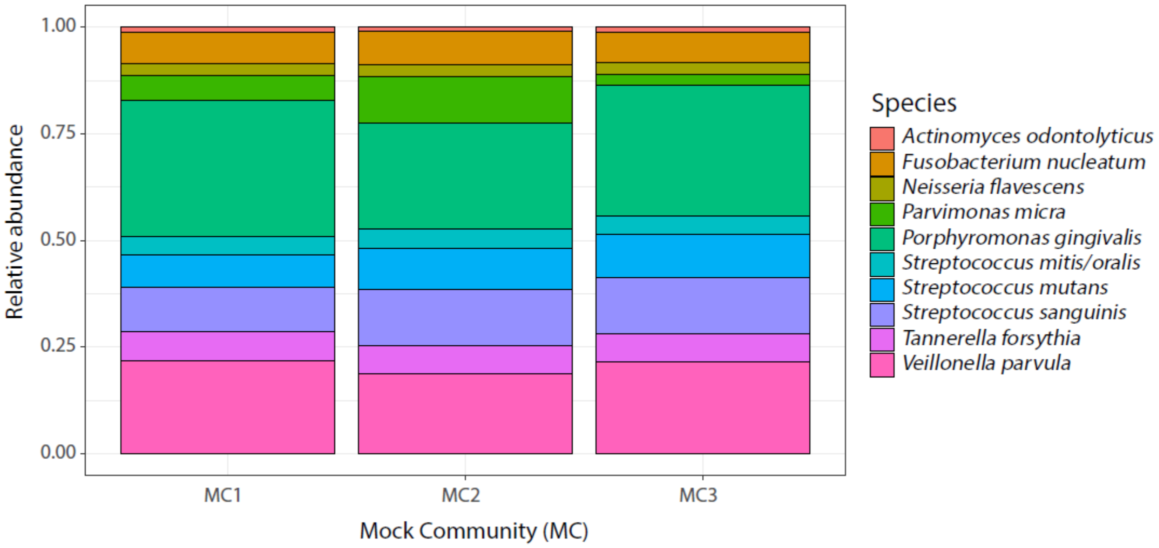

Supplementary -Figure 1: Mock community in triplicate.

Supplementary Figures

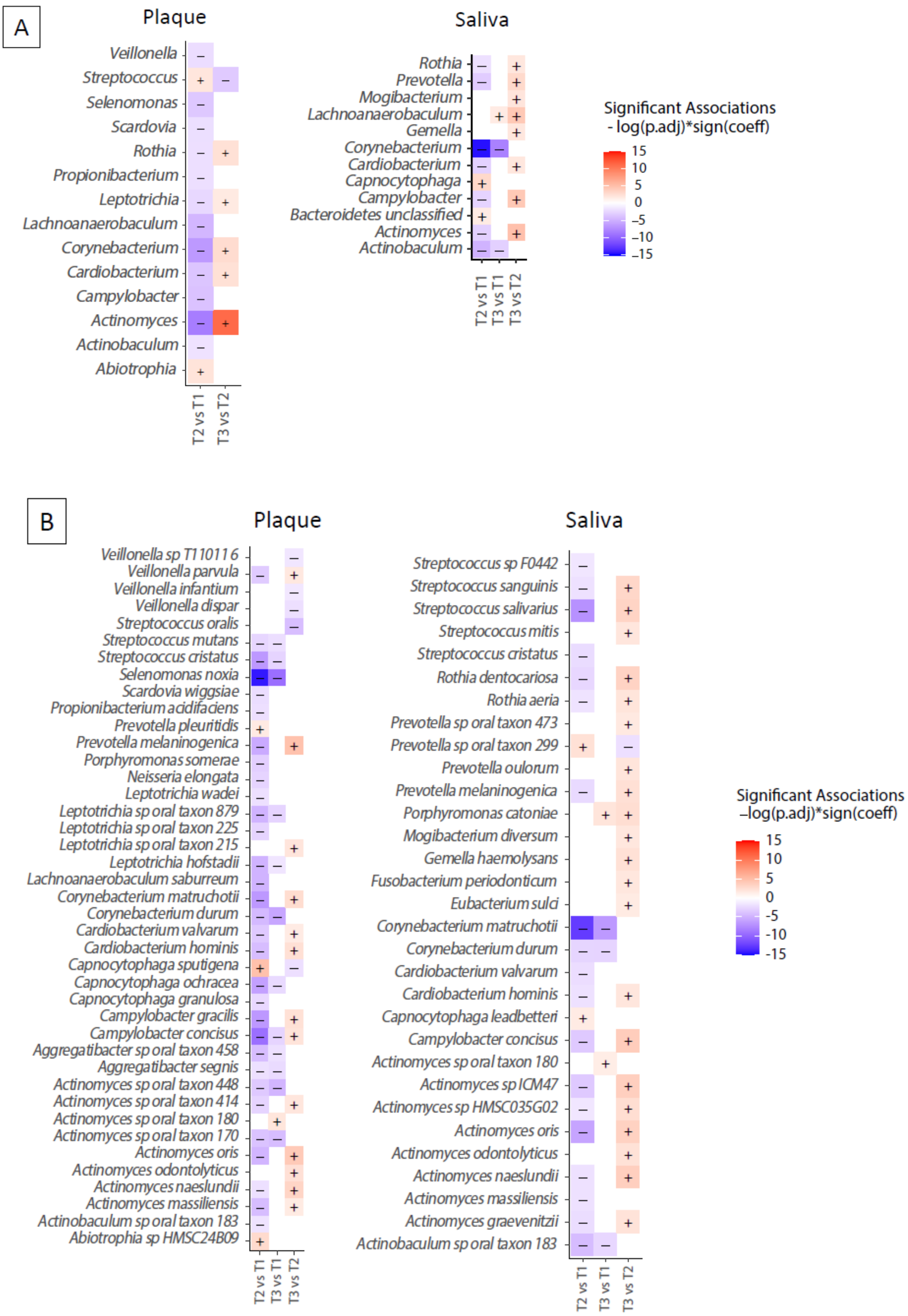

Supplementary-Figure 2: All taxa with significant changes in abundance (without a threshold of 1%) in supragingival plaque and saliva between the different timepoints. A: Genera; B: Species.

Supplementary Tables

Supplementary-Table 1: Overview of the patient characteristics and the procedures involved in this study. Smoker: 1 – yes, 0 – no. CPITN: The community periodontal index of treatment needs. For CPITN, the dentition was divided into six sextants. 0 = healthy periodontium; 1 = presence of bleeding on probing; 2 = presence of plaque or calculus; 3 = pocket probing depths between 4 and 5 mm; 4 = pocket probing depths higher than 5 mm.

Patient no.	Sex	Age [years]	Smoker	Pack years (smoker)	CPITN	Saliva flow rate [ml/min]	Saliva pH; without stimulation	Saliva buffer capacity
1	m	38	0		3,3,2,4,4,3	2	7.5	6
2	drop out							
3	m	18	0		0,0,1,0,0,0	2.5	7.5	6.5
4	m	29	0		1,1,1,1,1,1	2.3	7.5	5
5	f	27	0		0,0,0,0,1,0	2	7.5	7
6	f	22	1	1.25	0,0,1,0,2,0	1.2	7	5.5
7	m	71	0		1,1,4,1,2,1	2.2	7	5
8	f	18	0		0,0,0,0,1,0	1.2	7.5	6.5
9	f	20	0		0,1,0,0,1,0	1	7.5	6.5
10	f	79	0		1,4,3,1,2,3	2.1	7	5
11	f	56	0		3,2,3,2,2,2	1.3	7.5	6.5
12	f	35	0		0,0,0,0,0,0	2	7.5	6
13	m	50	0		4,3,4,3,4,3	3.6	7	5.5
14	f	25	0		0,0,0,1,1,0	1.7	7.5	6.5
15	f	70	1	13.25	- , - , - , - , 4, -	2	7.5	6.5
16	f	44	0		4,1,1,1,2,1	1	7	5.5
17	f	23	0		0,0,0,0,0,0	1.4	7.5	5.5
18	m	56	0		3,1,4,1,2,1	1.6	7.5	6.5
19	f	39	0		0,0,0,0,1,0	1.4	7.5	6
20	f	27	0		0,0,1,0,0,0	1.2	6.5	5.5
21	m	71	0		0,0,4,0,0,0	1.3	7.5	7

Supplementary Tables

Supplementary-Table 2: Median relative abundance and standard deviation of genera and species in plaque and saliva.

Genus supragingival plaque	T1	T1_sd	T2	T2_sd	T3	T3_sd
Brochothrix	0.0000	0.0000	0.0000	0.0000	0.0000	0.0000
Abiotrophia	0.0000	0.0006	0.0012	0.0074	0.0000	0.0023
Gemella	0.0030	0.0141	0.0038	0.0027	0.0026	0.0066
Catonella	0.0001	0.0016	0.0002	0.0021	0.0006	0.0011
Lactococcus	0.0000	0.0001	0.0000	0.0000	0.0000	0.0000
Enterococcus	0.0000	0.0000	0.0000	0.0180	0.0000	0.0000
Johnsonella	0.0000	0.0007	0.0000	0.0007	0.0000	0.0010
Leuconostoc	0.0000	0.0000	0.0000	0.0000	0.0000	0.0000
Oribacterium	0.0013	0.0023	0.0016	0.0042	0.0009	0.0046
Lactobacillus	0.0000	0.0005	0.0000	0.0000	0.0000	0.0000
Streptococcus	0.0817	0.1032	0.2576	0.1688	0.0753	0.0837
Prevotella	0.0682	0.0669	0.0267	0.0430	0.0366	0.0353
Rothia	0.0543	0.0726	0.0181	0.1891	0.0539	0.2278
Dialister	0.0015	0.0050	0.0010	0.0045	0.0008	0.0024
Tannerella	0.0041	0.0137	0.0053	0.0197	0.0073	0.0214
Shuttleworthia	0.0000	0.0018	0.0000	0.0008	0.0000	0.0008
Stomatobaculum	0.0000	0.0023	0.0001	0.0009	0.0000	0.0008
Bacteroides	0.0000	0.0000	0.0000	0.0002	0.0000	0.0000
Parvimonas	0.0011	0.0031	0.0019	0.0062	0.0019	0.0025
Treponema	0.0022	0.0086	0.0037	0.0225	0.0023	0.0076
Centipeda	0.0000	0.0008	0.0000	0.0001	0.0000	0.0003
Pseudoramibacter	0.0000	0.0005	0.0000	0.0001	0.0000	0.0010
Megasphaera	0.0000	0.0035	0.0000	0.0004	0.0000	0.0005
Veillonella	0.0502	0.0678	0.0214	0.0671	0.0274	0.0354
Granulicatella	0.0001	0.0006	0.0004	0.0035	0.0001	0.0003
Slackia	0.0000	0.0014	0.0001	0.0018	0.0000	0.0005
Anaeroglobus	0.0002	0.0047	0.0001	0.0032	0.0006	0.0026
Filifactor	0.0000	0.0010	0.0000	0.0165	0.0000	0.0009
Alloprevotella	0.0020	0.0178	0.0005	0.0147	0.0014	0.0187
Pyramidobacter	0.0000	0.0000	0.0000	0.0000	0.0000	0.0000
Mitsuokella	0.0000	0.0002	0.0000	0.0000	0.0000	0.0000
Selenomonas	0.0117	0.0123	0.0036	0.0139	0.0048	0.0086
Fretibacterium	0.0000	0.0009	0.0003	0.0026	0.0001	0.0015
Olsenella	0.0001	0.0018	0.0000	0.0045	0.0000	0.0040
Atopobium	0.0028	0.0241	0.0025	0.0205	0.0038	0.0257
Kingella	0.0035	0.0065	0.0052	0.0104	0.0051	0.0075
Eikenella	0.0011	0.0031	0.0010	0.0099	0.0009	0.0020
Neisseria	0.0202	0.0553	0.0360	0.1426	0.0137	0.0371
Micrococcus	0.0000	0.0000	0.0000	0.0000	0.0000	0.0000
Candida	0.0000	0.0000	0.0000	0.0000	0.0000	0.0000
Lachnoanaerobaculum	0.0023	0.0053	0.0000	0.0017	0.0003	0.0038

Morococcus	0.0000	0.0001	0.0000	0.0000	0.0000	0.0000
Porphyromonas	0.0050	0.0061	0.0032	0.0379	0.0015	0.0114
Leptotrichia	0.0191	0.0315	0.0022	0.0121	0.0063	0.0215
Ottowia	0.0000	0.0014	0.0000	0.0130	0.0000	0.0015
Simonsiella	0.0000	0.0000	0.0000	0.0000	0.0000	0.0000
Fusobacterium	0.0145	0.0087	0.0151	0.0205	0.0078	0.0092
Actinomyces	0.1947	0.0955	0.0402	0.0622	0.3854	0.2272
Peptoanaerobacter	0.0000	0.0009	0.0005	0.0017	0.0002	0.0010
Lautropia	0.0024	0.0249	0.0303	0.0463	0.0055	0.0253
Peptostreptococcus	0.0000	0.0023	0.0000	0.0049	0.0001	0.0012
Scardovia	0.0004	0.0447	0.0000	0.0024	0.0000	0.0058
Actinobaculum	0.0353	0.0489	0.0049	0.0175	0.0059	0.0126
Eggerthia	0.0000	0.0050	0.0000	0.0122	0.0000	0.0094
Bulleidia	0.0001	0.0011	0.0002	0.0026	0.0002	0.0008
Haemophilus	0.0025	0.0090	0.0042	0.1061	0.0013	0.0077
Gardnerella	0.0000	0.0000	0.0000	0.0000	0.0000	0.0000
Capnocytophaga	0.0244	0.0185	0.0459	0.0690	0.0216	0.0210
Parascardovia	0.0000	0.0009	0.0000	0.0002	0.0000	0.0000
*Lachnospiraceae*_unclassified	0.0000	0.0000	0.0000	0.0000	0.0000	0.0000
Alloscardovia	0.0000	0.0000	0.0000	0.0000	0.0000	0.0000
Solobacterium	0.0016	0.0022	0.0006	0.0031	0.0004	0.0017
Aggregatibacter	0.0024	0.0084	0.0003	0.0110	0.0001	0.0037
Bifidobacterium	0.0000	0.0053	0.0000	0.0062	0.0000	0.0034
Corynebacterium	0.0563	0.0826	0.0014	0.0131	0.0392	0.0485
Bilophila	0.0000	0.0001	0.0000	0.0000	0.0000	0.0000
Desulfobulbus	0.0000	0.0008	0.0000	0.0012	0.0000	0.0014
*Anaerolineaceae*_unclassified	0.0000	0.0002	0.0000	0.0009	0.0000	0.0001
Cutibacterium	0.0000	0.0000	0.0000	0.0000	0.0000	0.0000
Cardiobacterium	0.0098	0.0170	0.0002	0.0051	0.0052	0.0224
Campylobacter	0.0097	0.0063	0.0035	0.0088	0.0061	0.0036
Propionibacterium	0.0003	0.0265	0.0000	0.0208	0.0000	0.0018
Mogibacterium	0.0000	0.0012	0.0000	0.0008	0.0002	0.0014
*Peptostreptococcaceae*_unclassified	0.0000	0.0001	0.0000	0.0000	0.0000	0.0001
Pseudopropionibacterium	0.0021	0.0173	0.0063	0.0097	0.0062	0.0133
*Bacteroidetes*_unclassified	0.0001	0.0018	0.0005	0.0102	0.0000	0.0027
*Clostridiales_Family_XIII_Incertae_Sedis*_unclassified	0.0044	0.0123	0.0061	0.0222	0.0054	0.0084

**Species supragingival plaque**	**T1**	**T1_sd**	**T2**	**T2_sd**	**T3**	**T3_sd**
Candida_dubliniensis	0.0000	0.0000	0.0000	0.0000	0.0000	0.0000
Fretibacterium_fastidiosum	0.0000	0.0009	0.0003	0.0026	0.0001	0.0015
Pyramidobacter_piscolens	0.0000	0.0000	0.0000	0.0000	0.0000	0.0000
Leptotrichia_goodfellowii	0.0000	0.0004	0.0000	0.0009	0.0000	0.0002
*Leptotrichia*_sp_oral_taxon_215	0.0000	0.0020	0.0000	0.0002	0.0000	0.0005
*Leptotrichia*_sp_oral_taxon_212	0.0006	0.0024	0.0001	0.0028	0.0006	0.0032

*Leptotrichia*_sp_oral_taxon_847	0.0000	0.0001	0.0000	0.0004	0.0000	0.0001
*Leptotrichia*_sp_oral_taxon_498	0.0000	0.0088	0.0000	0.0052	0.0000	0.0027
Leptotrichia_wadei	0.0037	0.0089	0.0002	0.0061	0.0012	0.0184
Leptotrichia_buccalis	0.0001	0.0056	0.0000	0.0012	0.0000	0.0033
Leptotrichia_hofstadii	0.0018	0.0155	0.0004	0.0013	0.0007	0.0026
*Leptotrichia*_sp_oral_taxon_879	0.0007	0.0029	0.0000	0.0004	0.0000	0.0003
*Leptotrichia*_sp_oral_taxon_225	0.0006	0.0028	0.0000	0.0006	0.0001	0.0007
Fusobacterium_necrophorum	0.0000	0.0000	0.0000	0.0006	0.0000	0.0000
*Fusobacterium*_sp_oral_taxon_370	0.0000	0.0005	0.0000	0.0011	0.0000	0.0001
Fusobacterium_periodonticum	0.0000	0.0004	0.0000	0.0007	0.0000	0.0002
Fusobacterium_nucleatum	0.0129	0.0088	0.0123	0.0196	0.0064	0.0093
Fusobacterium_hwasookii	0.0000	0.0004	0.0000	0.0031	0.0000	0.0015
Campylobacter_rectus	0.0000	0.0004	0.0000	0.0052	0.0000	0.0003
Campylobacter_showae	0.0008	0.0025	0.0016	0.0035	0.0007	0.0021
Campylobacter_curvus	0.0000	0.0001	0.0000	0.0001	0.0000	0.0000
Campylobacter_concisus	0.0006	0.0028	0.0000	0.0003	0.0002	0.0009
Campylobacter_gracilis	0.0059	0.0043	0.0013	0.0024	0.0029	0.0029
Treponema_denticola	0.0000	0.0048	0.0001	0.0131	0.0000	0.0048
Treponema_vincentii	0.0000	0.0007	0.0000	0.0007	0.0000	0.0009
Treponema_medium	0.0000	0.0001	0.0000	0.0006	0.0000	0.0002
*Treponema*_sp_OMZ_838	0.0000	0.0008	0.0001	0.0012	0.0000	0.0004
Treponema_maltophilum	0.0001	0.0014	0.0005	0.0016	0.0002	0.0005
Treponema_lecithinolyticum	0.0000	0.0004	0.0000	0.0025	0.0000	0.0014
Treponema_socranskii	0.0011	0.0018	0.0007	0.0044	0.0015	0.0022
*Anaerolineaceae*_bacterium_oral_taxon_439	0.0000	0.0002	0.0000	0.0009	0.0000	0.0001
Corynebacterium_durum	0.0007	0.0088	0.0000	0.0014	0.0000	0.0005
Corynebacterium_matruchotii	0.0534	0.0760	0.0014	0.0127	0.0392	0.0484
Pseudopropionibacterium_propionicum	0.0021	0.0173	0.0063	0.0097	0.0062	0.0133
Propionibacterium_acidifaciens	0.0003	0.0265	0.0000	0.0208	0.0000	0.0018
Cutibacterium_acnes	0.0000	0.0000	0.0000	0.0000	0.0000	0.0000
*Actinobaculum*_sp_oral_taxon_183	0.0353	0.0489	0.0049	0.0175	0.0059	0.0126
Actinomyces_graevenitzii	0.0000	0.0003	0.0000	0.0005	0.0000	0.0006
*Actinomyces*_sp_oral_taxon_897	0.0006	0.0112	0.0000	0.0136	0.0001	0.0173
Actinomyces_johnsonii	0.0004	0.0088	0.0001	0.0026	0.0000	0.0084
Actinomyces_viscosus	0.0000	0.0084	0.0000	0.0008	0.0000	0.0140
Actinomyces_naeslundii	0.0660	0.0750	0.0036	0.0427	0.1876	0.2182
*Actinomyces*_sp_oral_taxon_170	0.0000	0.0062	0.0000	0.0002	0.0000	0.0000
Actinomyces_oris	0.0262	0.0395	0.0028	0.0078	0.0112	0.0568
*Actinomyces*_sp_oral_taxon_414	0.0079	0.0171	0.0027	0.0062	0.0058	0.0087
Actinomyces_massiliensis	0.0052	0.0171	0.0002	0.0018	0.0015	0.0138
*Actinomyces*_sp_oral_taxon_448	0.0109	0.0620	0.0009	0.0042	0.0001	0.0281
Actinomyces_turicensis	0.0000	0.0000	0.0000	0.0002	0.0000	0.0001
Actinomyces_cardiffensis	0.0000	0.0005	0.0000	0.0001	0.0000	0.0008
*Actinomyces*_sp_oral_taxon_181	0.0000	0.0016	0.0000	0.0003	0.0000	0.0014

*Actinomyces*_sp_S6_Spd3	0.0000	0.0000	0.0000	0.0001	0.0000	0.0000
Actinomyces_georgiae	0.0005	0.0010	0.0007	0.0012	0.0006	0.0027
Actinomyces_hongkongensis	0.0003	0.0018	0.0000	0.0013	0.0004	0.0120
Actinomyces_meyeri	0.0000	0.0004	0.0002	0.0036	0.0000	0.0005
*Actinomyces*_sp_ICM47	0.0000	0.0001	0.0000	0.0001	0.0000	0.0009
Actinomyces_odontolyticus	0.0002	0.0090	0.0000	0.0200	0.0007	0.0106
*Actinomyces*_sp_HMSC035G02	0.0000	0.0003	0.0000	0.0003	0.0000	0.0004
*Actinomyces*_sp_oral_taxon_180	0.0009	0.0068	0.0015	0.0143	0.0062	0.0300
*Actinomyces*_sp_HPA0247	0.0000	0.0003	0.0000	0.0004	0.0000	0.0002
Alloscardovia_omnicolens	0.0000	0.0000	0.0000	0.0000	0.0000	0.0000
Parascardovia_denticolens	0.0000	0.0009	0.0000	0.0002	0.0000	0.0000
Scardovia_wiggsiae	0.0004	0.0447	0.0000	0.0024	0.0000	0.0058
Gardnerella_vaginalis	0.0000	0.0000	0.0000	0.0000	0.0000	0.0000
Bifidobacterium_scardovii	0.0000	0.0000	0.0000	0.0000	0.0000	0.0001
Bifidobacterium_dentium	0.0000	0.0053	0.0000	0.0062	0.0000	0.0033
Bifidobacterium_longum	0.0000	0.0000	0.0000	0.0000	0.0000	0.0000
Micrococcus_luteus	0.0000	0.0000	0.0000	0.0000	0.0000	0.0000
Rothia_aeria	0.0090	0.0475	0.0017	0.0198	0.0091	0.0502
Rothia_mucilaginosa	0.0003	0.0120	0.0001	0.0396	0.0003	0.0021
Rothia_dentocariosa	0.0407	0.0505	0.0062	0.1831	0.0148	0.2309
Slackia_exigua	0.0000	0.0014	0.0001	0.0018	0.0000	0.0005
Atopobium_minutum	0.0000	0.0000	0.0000	0.0000	0.0000	0.0000
Atopobium_parvulum	0.0004	0.0011	0.0003	0.0013	0.0004	0.0039
Atopobium_rimae	0.0016	0.0238	0.0017	0.0196	0.0019	0.0235
Olsenella_uli	0.0000	0.0017	0.0000	0.0045	0.0000	0.0036
Olsenella_scatoligenes	0.0000	0.0000	0.0000	0.0000	0.0000	0.0000
Dialister_micraerophilus	0.0000	0.0002	0.0000	0.0000	0.0000	0.0000
Dialister_pneumosintes	0.0000	0.0017	0.0001	0.0044	0.0001	0.0012
Dialister_invisus	0.0006	0.0049	0.0002	0.0016	0.0002	0.0016
Megasphaera_micronuciformis	0.0000	0.0035	0.0000	0.0004	0.0000	0.0005
Anaeroglobus_geminatus	0.0002	0.0047	0.0001	0.0032	0.0006	0.0026
Veillonella_atypica	0.0000	0.0004	0.0000	0.0077	0.0000	0.0007
Veillonella_tobetsuensis	0.0000	0.0000	0.0000	0.0000	0.0000	0.0000
Veillonella_parvula	0.0497	0.0671	0.0124	0.0653	0.0274	0.0353
Veillonella_rodentium	0.0000	0.0001	0.0000	0.0000	0.0000	0.0000
*Veillonella*_sp_T11011_6	0.0000	0.0001	0.0000	0.0004	0.0000	0.0000
Veillonella_infantium	0.0000	0.0001	0.0000	0.0004	0.0000	0.0000
Veillonella_dispar	0.0000	0.0005	0.0001	0.0053	0.0000	0.0002
Veillonella_rogosae	0.0000	0.0000	0.0000	0.0001	0.0000	0.0000
*Mitsuokella*_sp_oral_taxon_131	0.0000	0.0002	0.0000	0.0000	0.0000	0.0000
Selenomonas_sputigena	0.0009	0.0032	0.0007	0.0048	0.0015	0.0016
Selenomonas_infelix	0.0001	0.0013	0.0000	0.0004	0.0000	0.0003
*Selenomonas*_sp_oral_taxon_920	0.0001	0.0032	0.0000	0.0013	0.0002	0.0010
*Selenomonas*_sp_FOBRC6	0.0001	0.0013	0.0000	0.0012	0.0002	0.0022
*Selenomonas*_sp_oral_taxon_138	0.0000	0.0003	0.0000	0.0033	0.0000	0.0002
*Selenomonas*_sp_oral_taxon_892	0.0000	0.0001	0.0000	0.0001	0.0000	0.0004

Selenomonas_noxia	0.0042	0.0106	0.0000	0.0007	0.0000	0.0059
Selenomonas_artemidis	0.0003	0.0014	0.0007	0.0017	0.0011	0.0018
Selenomonas_flueggei	0.0000	0.0005	0.0000	0.0003	0.0001	0.0005
Centipeda_periodontii	0.0000	0.0008	0.0000	0.0001	0.0000	0.0003
*Selenomonas*_sp_F0473	0.0000	0.0000	0.0000	0.0000	0.0000	0.0000
*Selenomonas*_sp_oral_taxon_126	0.0000	0.0013	0.0000	0.0069	0.0000	0.0013
Pseudoramibacter_alactolyticus	0.0000	0.0005	0.0000	0.0001	0.0000	0.0010
*Peptostreptococcaceae*_bacterium_oral_taxon_113	0.0000	0.0001	0.0000	0.0000	0.0000	0.0001
*Parvimonas*_sp_oral_taxon_110	0.0000	0.0007	0.0000	0.0020	0.0000	0.0003
*Parvimonas*_sp_oral_taxon_393	0.0000	0.0001	0.0000	0.0008	0.0000	0.0000
Parvimonas_micra	0.0011	0.0029	0.0013	0.0060	0.0019	0.0025
Filifactor_alocis	0.0000	0.0010	0.0000	0.0165	0.0000	0.0009
Peptoanaerobacter_stomatis	0.0000	0.0006	0.0000	0.0011	0.0000	0.0001
Eubacterium_yurii	0.0000	0.0007	0.0003	0.0015	0.0001	0.0010
Peptostreptococcus_stomatis	0.0000	0.0023	0.0000	0.0049	0.0001	0.0012
Eubacterium_saphenum	0.0000	0.0002	0.0000	0.0002	0.0000	0.0005
Eubacterium_brachy	0.0020	0.0110	0.0030	0.0191	0.0051	0.0066
Mogibacterium_timidum	0.0000	0.0012	0.0000	0.0007	0.0000	0.0014
Mogibacterium_pumilum	0.0000	0.0000	0.0000	0.0000	0.0000	0.0000
Mogibacterium_diversum	0.0000	0.0001	0.0000	0.0004	0.0001	0.0003
Eubacterium_sulci	0.0000	0.0002	0.0000	0.0000	0.0000	0.0002
Eubacterium_infirmum	0.0001	0.0021	0.0004	0.0043	0.0001	0.0023
Eubacterium_nodatum	0.0000	0.0007	0.0000	0.0006	0.0000	0.0029
Eubacterium_minutum	0.0000	0.0002	0.0000	0.0025	0.0000	0.0002
Catonella_morbi	0.0001	0.0016	0.0002	0.0021	0.0006	0.0011
*Lachnospiraceae*_bacterium_oral_taxon_096	0.0000	0.0000	0.0000	0.0000	0.0000	0.0000
Johnsonella_ignava	0.0000	0.0007	0.0000	0.0007	0.0000	0.0010
Lachnoanaerobaculum_saburreum	0.0023	0.0053	0.0000	0.0017	0.0003	0.0038
Lachnoanaerobaculum_umeaense	0.0000	0.0000	0.0000	0.0000	0.0000	0.0000
Stomatobaculum_longum	0.0000	0.0023	0.0001	0.0009	0.0000	0.0008
*Oribacterium*_sp_oral_taxon_078	0.0012	0.0023	0.0016	0.0041	0.0009	0.0046
Oribacterium_sinus	0.0000	0.0001	0.0000	0.0000	0.0000	0.0000
Oribacterium_asaccharolyticum	0.0000	0.0000	0.0000	0.0000	0.0000	0.0000
Oribacterium_parvum	0.0000	0.0000	0.0000	0.0000	0.0000	0.0000
Shuttleworthia_satelles	0.0000	0.0018	0.0000	0.0008	0.0000	0.0008
Brochothrix_thermosphacta	0.0000	0.0000	0.0000	0.0000	0.0000	0.0000
Eggerthia_catenaformis	0.0000	0.0050	0.0000	0.0122	0.0000	0.0094
Solobacterium_moorei	0.0016	0.0022	0.0006	0.0031	0.0004	0.0017
Gemella_morbillorum	0.0018	0.0134	0.0030	0.0023	0.0023	0.0058
Gemella_sanguinis	0.0002	0.0007	0.0000	0.0014	0.0002	0.0005
Gemella_haemolysans	0.0000	0.0005	0.0000	0.0001	0.0000	0.0003
Abiotrophia_defectiva	0.0000	0.0002	0.0000	0.0027	0.0000	0.0005
*Abiotrophia*_sp_HMSC24B09	0.0000	0.0004	0.0009	0.0050	0.0000	0.0019
Granulicatella_adiacens	0.0001	0.0006	0.0003	0.0035	0.0001	0.0003

Enterococcus_faecalis	0.0000	0.0000	0.0000	0.0180	0.0000	0.0000
Lactococcus_lactis	0.0000	0.0001	0.0000	0.0000	0.0000	0.0000
Streptococcus_peroris	0.0000	0.0000	0.0000	0.0001	0.0000	0.0000
Streptococcus_infantis	0.0001	0.0027	0.0000	0.0105	0.0000	0.0010
Streptococcus_cristatus	0.0024	0.0086	0.0000	0.0008	0.0000	0.0054
Streptococcus_sanguinis	0.0161	0.0304	0.0214	0.1204	0.0258	0.0447
*Streptococcus*_sp_oral_taxon_056	0.0000	0.0002	0.0000	0.0013	0.0000	0.0016
*Streptococcus*_sp_F0442	0.0000	0.0001	0.0000	0.0000	0.0000	0.0000
*Streptococcus*_sp_A12	0.0000	0.0004	0.0000	0.0003	0.0000	0.0001
Streptococcus_australis	0.0000	0.0001	0.0000	0.0000	0.0000	0.0000
Streptococcus_gordonii	0.0020	0.0318	0.0003	0.1344	0.0019	0.0199
Streptococcus_milleri	0.0000	0.0001	0.0000	0.0004	0.0000	0.0015
Streptococcus_anginosus_group	0.0019	0.0136	0.0006	0.0070	0.0009	0.0224
Streptococcus_viridans	0.0000	0.0027	0.0000	0.0020	0.0000	0.0292
Streptococcus_parasanguinis	0.0000	0.0011	0.0002	0.0172	0.0001	0.0015
*Streptococcus*_sp_HMSC034E03	0.0000	0.0000	0.0000	0.0000	0.0000	0.0000
*Streptococcus*_sp_HMSC071D03	0.0000	0.0000	0.0000	0.0000	0.0000	0.0000
*Streptococcus*_sp_HMSC067H01	0.0000	0.0000	0.0000	0.0000	0.0000	0.0000
*Streptococcus*_sp_HPH0090	0.0000	0.0000	0.0000	0.0000	0.0000	0.0000
Streptococcus_mitis	0.0001	0.0200	0.0000	0.0049	0.0002	0.0023
*Streptococcus*_sp_M334	0.0000	0.0000	0.0000	0.0000	0.0000	0.0000
Streptococcus_pseudopneumoniae	0.0000	0.0000	0.0000	0.0000	0.0000	0.0000
*Streptococcus*_sp_HMSC070B10	0.0000	0.0000	0.0000	0.0000	0.0000	0.0000
*Streptococcus*_sp_oral_taxon_058	0.0000	0.0001	0.0000	0.0002	0.0000	0.0001
Streptococcus_oralis	0.0206	0.0512	0.0551	0.1319	0.0186	0.0157
*Streptococcus*_sp_NLAE_zl_C503	0.0000	0.0069	0.0000	0.0020	0.0000	0.0039
Streptococcus_mutans	0.0001	0.0174	0.0000	0.0007	0.0000	0.0029
Streptococcus_sobrinus	0.0000	0.0027	0.0000	0.0000	0.0000	0.0000
Streptococcus_thermophilus	0.0000	0.0001	0.0000	0.0000	0.0000	0.0000
Streptococcus_vestibularis	0.0000	0.0000	0.0000	0.0000	0.0000	0.0000
*Streptococcus_salivarius*_CAG_79	0.0000	0.0000	0.0000	0.0000	0.0000	0.0000
Streptococcus_salivarius	0.0000	0.0002	0.0000	0.0002	0.0000	0.0006
Leuconostoc_carnosum	0.0000	0.0000	0.0000	0.0000	0.0000	0.0000
Lactobacillus_rhamnosus	0.0000	0.0001	0.0000	0.0000	0.0000	0.0000
Lactobacillus_kalixensis	0.0000	0.0000	0.0000	0.0000	0.0000	0.0000
Lactobacillus_gasseri	0.0000	0.0000	0.0000	0.0000	0.0000	0.0000
Lactobacillus_salivarius	0.0000	0.0000	0.0000	0.0000	0.0000	0.0000
Lactobacillus_fermentum	0.0000	0.0005	0.0000	0.0000	0.0000	0.0000
Lactobacillus_vaginalis	0.0000	0.0001	0.0000	0.0000	0.0000	0.0000
*Bacteroidetes*_oral_taxon_274	0.0001	0.0015	0.0005	0.0102	0.0000	0.0026
Tannerella_forsythia	0.0000	0.0012	0.0004	0.0068	0.0001	0.0024
*Tannerella*_sp_oral_taxon_HOT_286	0.0026	0.0115	0.0039	0.0182	0.0048	0.0215
*Tannerella*_sp_oral_taxon_808	0.0005	0.0028	0.0001	0.0030	0.0000	0.0012
Porphyromonas_canoris	0.0000	0.0000	0.0000	0.0000	0.0000	0.0001
Porphyromonas_endodontalis	0.0000	0.0029	0.0003	0.0306	0.0001	0.0015
Porphyromonas_gingivalis	0.0000	0.0046	0.0000	0.0258	0.0000	0.0104

*Porphyromonas*_sp_oral_taxon_278	0.0000	0.0017	0.0000	0.0022	0.0000	0.0020
Porphyromonas_catoniae	0.0004	0.0022	0.0003	0.0016	0.0004	0.0016
Porphyromonas_somerae	0.0000	0.0016	0.0000	0.0001	0.0000	0.0002
*Bacteroidetes*_bacterium_oral_taxon _272	0.0000	0.0008	0.0000	0.0000	0.0000	0.0003
Bacteroides_heparinolyticus	0.0000	0.0000	0.0000	0.0002	0.0000	0.0000
*Prevotella*_sp_oral_taxon_473	0.0000	0.0004	0.0000	0.0012	0.0000	0.0008
Alloprevotella_tannerae	0.0014	0.0176	0.0005	0.0144	0.0010	0.0186
Alloprevotella_rava	0.0000	0.0001	0.0000	0.0000	0.0000	0.0000
Prevotella_pleuritidis	0.0000	0.0009	0.0000	0.0086	0.0000	0.0005
Prevotella_enoeca	0.0000	0.0002	0.0000	0.0008	0.0000	0.0002
Prevotella_marshii	0.0000	0.0002	0.0000	0.0010	0.0000	0.0006
*Prevotella*_sp_oral_taxon_299	0.0000	0.0000	0.0000	0.0000	0.0000	0.0000
Prevotella_saccharolytica	0.0004	0.0012	0.0000	0.0007	0.0001	0.0006
Prevotella_loescheii	0.0000	0.0017	0.0006	0.0023	0.0004	0.0052
Prevotella_shahii	0.0000	0.0000	0.0000	0.0000	0.0000	0.0000
Prevotella_micans	0.0000	0.0034	0.0002	0.0037	0.0000	0.0016
Prevotella_multiformis	0.0000	0.0000	0.0000	0.0000	0.0000	0.0000
Prevotella_histicola	0.0000	0.0002	0.0000	0.0003	0.0000	0.0005
Prevotella_denticola	0.0028	0.0266	0.0000	0.0112	0.0012	0.0122
Prevotella_veroralis	0.0000	0.0015	0.0000	0.0010	0.0000	0.0013
*Prevotella*_sp_oral_taxon_306	0.0000	0.0004	0.0000	0.0011	0.0000	0.0004
Prevotella_fusca	0.0000	0.0006	0.0000	0.0003	0.0000	0.0001
Prevotella_scopos	0.0000	0.0000	0.0000	0.0000	0.0000	0.0000
*Prevotella*_sp_F0091	0.0000	0.0011	0.0000	0.0001	0.0000	0.0005
Prevotella_melaninogenica	0.0003	0.0039	0.0000	0.0002	0.0008	0.0026
Prevotella_jejuni	0.0000	0.0001	0.0000	0.0004	0.0000	0.0003
Prevotella_intermedia	0.0000	0.0099	0.0000	0.0038	0.0000	0.0051
Prevotella_nigrescens	0.0170	0.0276	0.0152	0.0215	0.0069	0.0182
Prevotella_pallens	0.0000	0.0005	0.0000	0.0008	0.0000	0.0002
Prevotella_oulorum	0.0006	0.0052	0.0003	0.0025	0.0012	0.0060
Prevotella_baroniae	0.0000	0.0015	0.0000	0.0004	0.0000	0.0007
Prevotella_dentalis	0.0000	0.0009	0.0000	0.0011	0.0000	0.0005
Prevotella_buccae	0.0000	0.0024	0.0000	0.0022	0.0000	0.0011
Prevotella_oralis	0.0000	0.0040	0.0000	0.0012	0.0000	0.0016
*Prevotella*_sp_oral_taxon_376	0.0000	0.0020	0.0000	0.0004	0.0000	0.0003
Prevotella_oris	0.0110	0.0159	0.0029	0.0089	0.0030	0.0106
Prevotella_maculosa	0.0008	0.0025	0.0002	0.0010	0.0004	0.0015
Prevotella_salivae	0.0000	0.0035	0.0000	0.0006	0.0000	0.0022
Capnocytophaga_haemolytica	0.0000	0.0010	0.0000	0.0005	0.0000	0.0019
Capnocytophaga_ochracea	0.0076	0.0061	0.0013	0.0033	0.0027	0.0058
Capnocytophaga_sputigena	0.0038	0.0050	0.0185	0.0613	0.0041	0.0072
Capnocytophaga_leadbetteri	0.0026	0.0044	0.0077	0.0115	0.0025	0.0052
*Capnocytophaga*_sp_oral_taxon_332	0.0000	0.0005	0.0000	0.0013	0.0000	0.0008
Capnocytophaga_granulosa	0.0020	0.0065	0.0002	0.0006	0.0011	0.0039
*Capnocytophaga*_sp_oral_taxon_338	0.0000	0.0002	0.0000	0.0007	0.0000	0.0016

*Capnocytophaga*_sp_oral_taxon_863	0.0000	0.0001	0.0000	0.0001	0.0000	0.0000
Capnocytophaga_gingivalis	0.0039	0.0058	0.0022	0.0100	0.0050	0.0074
Desulfobulbus_oralis	0.0000	0.0008	0.0000	0.0012	0.0000	0.0014
Bilophila_wadsworthia	0.0000	0.0001	0.0000	0.0000	0.0000	0.0000
Neisseria_bacilliformis	0.0014	0.0088	0.0025	0.0333	0.0006	0.0166
Neisseria_elongata	0.0032	0.0068	0.0000	0.0244	0.0000	0.0060
Eikenella_corrodens	0.0010	0.0023	0.0010	0.0062	0.0008	0.0015
*Eikenella*_sp_NML130454	0.0000	0.0009	0.0000	0.0037	0.0001	0.0006
Kingella_denitrificans	0.0000	0.0040	0.0000	0.0000	0.0000	0.0033
Kingella_oralis	0.0032	0.0058	0.0052	0.0103	0.0037	0.0074
Simonsiella_muelleri	0.0000	0.0000	0.0000	0.0000	0.0000	0.0000
Neisseria_subflava	0.0000	0.0001	0.0000	0.0000	0.0000	0.0001
Neisseria_flavescens	0.0001	0.0124	0.0000	0.0014	0.0000	0.0026
Neisseria_perflava	0.0000	0.0000	0.0000	0.0000	0.0000	0.0000
Neisseria_cinerea	0.0000	0.0000	0.0000	0.0000	0.0000	0.0000
Morococcus_cerebrosus	0.0000	0.0001	0.0000	0.0000	0.0000	0.0000
Neisseria_sicca	0.0015	0.0370	0.0072	0.1301	0.0026	0.0127
*Neisseria*_sp_oral_taxon_014	0.0000	0.0376	0.0000	0.0138	0.0000	0.0150
*Neisseria*_sp_HMSC064E01	0.0000	0.0000	0.0000	0.0004	0.0000	0.0001
Neisseria_macacae	0.0000	0.0047	0.0000	0.0051	0.0000	0.0011
Neisseria_mucosa	0.0000	0.0026	0.0000	0.0178	0.0000	0.0037
Lautropia_mirabilis	0.0024	0.0249	0.0303	0.0463	0.0055	0.0253
*Ottowia*_sp_oral_taxon_894	0.0000	0.0014	0.0000	0.0130	0.0000	0.0015
Cardiobacterium_hominis	0.0044	0.0087	0.0000	0.0041	0.0021	0.0190
Cardiobacterium_valvarum	0.0019	0.0092	0.0001	0.0015	0.0007	0.0043
Haemophilus_parahaemolyticus	0.0000	0.0000	0.0000	0.0000	0.0000	0.0000
Haemophilus_paraphrohaemolyticus	0.0000	0.0000	0.0000	0.0000	0.0000	0.0000
Haemophilus_sputorum	0.0000	0.0002	0.0000	0.0003	0.0000	0.0000
Aggregatibacter_aphrophilus	0.0000	0.0023	0.0002	0.0103	0.0001	0.0029
Aggregatibacter_actinomycetemcomitans	0.0000	0.0000	0.0000	0.0000	0.0000	0.0004
*Aggregatibacter*_sp_oral_taxon_458	0.0004	0.0056	0.0000	0.0007	0.0000	0.0013
Aggregatibacter_segnis	0.0002	0.0017	0.0000	0.0015	0.0000	0.0012
Haemophilus_haemolyticus	0.0000	0.0000	0.0000	0.0000	0.0000	0.0000
Haemophilus_quentini	0.0000	0.0000	0.0000	0.0000	0.0000	0.0000
*Haemophilus*_sp_HMSC71H05	0.0000	0.0008	0.0000	0.0003	0.0000	0.0003
Haemophilus_parainfluenzae	0.0023	0.0090	0.0039	0.1061	0.0012	0.0077
Haemophilus_pittmaniae	0.0000	0.0000	0.0000	0.0000	0.0000	0.0000

**Genus saliva**	**T1**	**T1_sd**	**T2**	**T2_sd**	**T3**	**T3_sd**
Brochothrix	0.0000	0.0000	0.0000	0.0065	0.0000	0.0000
Abiotrophia	0.0000	0.0008	0.0000	0.0008	0.0000	0.0031
Gemella	0.0297	0.0286	0.0236	0.0277	0.0469	0.0363
Catonella	0.0000	0.0000	0.0000	0.0000	0.0000	0.0001
Lactococcus	0.0000	0.0007	0.0000	0.0000	0.0000	0.0000

Enterococcus	0.0000	0.0000	0.0000	0.0099	0.0000	0.0000
Johnsonella	0.0000	0.0000	0.0000	0.0000	0.0000	0.0000
Leuconostoc	0.0000	0.0000	0.0000	0.0004	0.0000	0.0000
Oribacterium	0.0006	0.0051	0.0010	0.0012	0.0010	0.0028
Lactobacillus	0.0000	0.0170	0.0000	0.0487	0.0000	0.0120
Streptococcus	0.2133	0.1289	0.2901	0.1602	0.2269	0.1180
Prevotella	0.0945	0.0980	0.0301	0.0834	0.0932	0.0798
Rothia	0.1609	0.1275	0.0679	0.1396	0.1261	0.0862
Dialister	0.0000	0.0005	0.0000	0.0004	0.0000	0.0004
Tannerella	0.0000	0.0040	0.0008	0.0026	0.0009	0.0045
Shuttleworthia	0.0000	0.0000	0.0000	0.0002	0.0000	0.0000
Stomatobaculum	0.0003	0.0024	0.0015	0.0044	0.0009	0.0031
Bacteroides	0.0000	0.0000	0.0000	0.0000	0.0000	0.0000
Parvimonas	0.0000	0.0014	0.0001	0.0059	0.0000	0.0023
Treponema	0.0000	0.0020	0.0000	0.0013	0.0000	0.0017
Centipeda	0.0000	0.0000	0.0000	0.0000	0.0000	0.0000
Pseudoramibacter	0.0000	0.0000	0.0000	0.0000	0.0000	0.0000
Megasphaera	0.0024	0.0159	0.0035	0.0129	0.0046	0.0132
Veillonella	0.0646	0.0606	0.0543	0.0506	0.0575	0.0570
Granulicatella	0.0001	0.0010	0.0002	0.0205	0.0004	0.0113
Slackia	0.0000	0.0000	0.0000	0.0001	0.0000	0.0000
Anaeroglobus	0.0000	0.0000	0.0000	0.0001	0.0000	0.0000
Filifactor	0.0000	0.0007	0.0000	0.0001	0.0000	0.0004
Alloprevotella	0.0012	0.0073	0.0014	0.0108	0.0055	0.0073
Pyramidobacter	0.0000	0.0000	0.0000	0.0000	0.0000	0.0000
Mitsuokella	0.0000	0.0000	0.0000	0.0000	0.0000	0.0000
Selenomonas	0.0000	0.0006	0.0000	0.0001	0.0000	0.0012
Fretibacterium	0.0000	0.0003	0.0000	0.0001	0.0000	0.0000
Olsenella	0.0000	0.0000	0.0000	0.0004	0.0000	0.0000
Atopobium	0.0019	0.0020	0.0021	0.0055	0.0025	0.0039
Kingella	0.0000	0.0015	0.0000	0.0011	0.0000	0.0008
Eikenella	0.0000	0.0011	0.0000	0.0022	0.0000	0.0007
Neisseria	0.0485	0.1090	0.0417	0.2096	0.0749	0.0994
Micrococcus	0.0000	0.0000	0.0000	0.0000	0.0000	0.0000
Candida	0.0000	0.0000	0.0000	0.0000	0.0000	0.0000
Lachnoanaerobaculum	0.0000	0.0013	0.0000	0.0018	0.0011	0.0017
Morococcus	0.0000	0.0001	0.0000	0.0000	0.0000	0.0019
Porphyromonas	0.0033	0.0193	0.0078	0.0237	0.0105	0.0211
Leptotrichia	0.0015	0.0044	0.0009	0.0027	0.0015	0.0071
Ottowia	0.0000	0.0010	0.0000	0.0005	0.0000	0.0001
Simonsiella	0.0000	0.0000	0.0000	0.0000	0.0000	0.0000
Fusobacterium	0.0042	0.0173	0.0033	0.0087	0.0056	0.0138
Actinomyces	0.1054	0.0629	0.0400	0.0842	0.1711	0.0867
Peptoanaerobacter	0.0000	0.0000	0.0000	0.0000	0.0000	0.0001
Lautropia	0.0016	0.0163	0.0003	0.0055	0.0024	0.0136
Peptostreptococcus	0.0000	0.0001	0.0000	0.0004	0.0000	0.0004

Scardovia	0.0000	0.0084	0.0000	0.0001	0.0000	0.0006
Actinobaculum	0.0000	0.0020	0.0000	0.0000	0.0000	0.0003
Eggerthia	0.0000	0.0005	0.0000	0.0001	0.0000	0.0008
Bulleidia	0.0000	0.0001	0.0000	0.0001	0.0000	0.0002
Haemophilus	0.0176	0.0256	0.0280	0.1083	0.0228	0.0199
Gardnerella	0.0000	0.0000	0.0000	0.0000	0.0000	0.0000
Capnocytophaga	0.0000	0.0131	0.0061	0.0594	0.0034	0.0067
Parascardovia	0.0000	0.0000	0.0000	0.0002	0.0000	0.0001
*Lachnospiraceae*_unclassified	0.0000	0.0020	0.0000	0.0010	0.0000	0.0013
Alloscardovia	0.0000	0.0066	0.0000	0.0003	0.0000	0.0003
Solobacterium	0.0000	0.0002	0.0000	0.0007	0.0002	0.0004
Aggregatibacter	0.0000	0.0024	0.0000	0.0048	0.0000	0.0015
Bifidobacterium	0.0000	0.0000	0.0000	0.0018	0.0000	0.0067
Corynebacterium	0.0024	0.0075	0.0000	0.0000	0.0000	0.0009
Bilophila	0.0000	0.0000	0.0000	0.0000	0.0000	0.0000
Desulfobulbus	0.0000	0.0003	0.0000	0.0000	0.0000	0.0001
*Anaerolineaceae*_unclassified	0.0000	0.0000	0.0000	0.0000	0.0000	0.0000
Cutibacterium	0.0000	0.0000	0.0000	0.0000	0.0000	0.0000
Cardiobacterium	0.0003	0.0037	0.0000	0.0004	0.0000	0.0019
Campylobacter	0.0048	0.0051	0.0009	0.0017	0.0047	0.0044
Propionibacterium	0.0000	0.0000	0.0000	0.0000	0.0000	0.0001
Mogibacterium	0.0015	0.0039	0.0002	0.0039	0.0024	0.0043
*Peptostreptococcaceae*_unclassified	0.0000	0.0000	0.0000	0.0000	0.0000	0.0000
Pseudopropionibacterium	0.0000	0.0020	0.0000	0.0004	0.0000	0.0023
Bacteroidetes_unclassified	0.0000	0.0000	0.0000	0.0006	0.0000	0.0002
*Clostridiales_Family_XIII_Incertae_Sedis*_unclassified	0.0021	0.0062	0.0008	0.0077	0.0043	0.0046

**Species saliva**	**T1**	**T1_sd**	**T2**	**T2_sd**	**T3**	**T3_sd**
Candida_dubliniensis	0.0000	0.0000	0.0000	0.0000	0.0000	0.0000
Fretibacterium_fastidiosum	0.0000	0.0003	0.0000	0.0001	0.0000	0.0000
Pyramidobacter_piscolens	0.0000	0.0000	0.0000	0.0000	0.0000	0.0000
Leptotrichia_goodfellowii	0.0000	0.0000	0.0000	0.0000	0.0000	0.0000
*Leptotrichia*_sp_oral_taxon_215	0.0000	0.0006	0.0000	0.0004	0.0000	0.0015
*Leptotrichia*_sp_oral_taxon_212	0.0000	0.0002	0.0000	0.0000	0.0000	0.0004
*Leptotrichia*_sp_oral_taxon_847	0.0000	0.0000	0.0000	0.0000	0.0000	0.0000
*Leptotrichia*_sp_oral_taxon_498	0.0000	0.0002	0.0000	0.0006	0.0000	0.0003
Leptotrichia_wadei	0.0009	0.0043	0.0004	0.0024	0.0002	0.0065
Leptotrichia_buccalis	0.0000	0.0000	0.0000	0.0000	0.0000	0.0000
Leptotrichia_hofstadii	0.0000	0.0002	0.0000	0.0000	0.0000	0.0002
*Leptotrichia*_sp_oral_taxon_879	0.0000	0.0000	0.0000	0.0000	0.0000	0.0000
*Leptotrichia*_sp_oral_taxon_225	0.0000	0.0000	0.0000	0.0000	0.0000	0.0001
Fusobacterium_necrophorum	0.0000	0.0000	0.0000	0.0003	0.0000	0.0000
*Fusobacterium*_sp_oral_taxon_370	0.0000	0.0000	0.0000	0.0000	0.0000	0.0000
Fusobacterium_periodonticum	0.0038	0.0170	0.0003	0.0065	0.0044	0.0138

Fusobacterium_nucleatum	0.0004	0.0025	0.0007	0.0036	0.0003	0.0012
Fusobacterium_hwasookii	0.0000	0.0002	0.0000	0.0000	0.0000	0.0005
Campylobacter_rectus	0.0000	0.0000	0.0000	0.0000	0.0000	0.0000
Campylobacter_showae	0.0000	0.0026	0.0000	0.0008	0.0000	0.0032
Campylobacter_curvus	0.0000	0.0000	0.0000	0.0000	0.0000	0.0000
Campylobacter_concisus	0.0039	0.0042	0.0003	0.0015	0.0021	0.0028
Campylobacter_gracilis	0.0000	0.0000	0.0000	0.0000	0.0000	0.0001
Treponema_denticola	0.0000	0.0016	0.0000	0.0013	0.0000	0.0006
Treponema_vincentii	0.0000	0.0000	0.0000	0.0000	0.0000	0.0000
Treponema_medium	0.0000	0.0000	0.0000	0.0000	0.0000	0.0000
*Treponema*_sp_OMZ_838	0.0000	0.0000	0.0000	0.0000	0.0000	0.0000
Treponema_maltophilum	0.0000	0.0000	0.0000	0.0000	0.0000	0.0000
Treponema_lecithinolyticum	0.0000	0.0012	0.0000	0.0002	0.0000	0.0011
Treponema_socranskii	0.0000	0.0000	0.0000	0.0002	0.0000	0.0000
*Anaerolineaceae_*bacterium_oral_taxon_439	0.0000	0.0000	0.0000	0.0000	0.0000	0.0000
Corynebacterium_durum	0.0000	0.0014	0.0000	0.0000	0.0000	0.0000
Corynebacterium_matruchotii	0.0020	0.0072	0.0000	0.0000	0.0000	0.0009
Pseudopropionibacterium_propionicum	0.0000	0.0020	0.0000	0.0004	0.0000	0.0023
Propionibacterium_acidifaciens	0.0000	0.0000	0.0000	0.0000	0.0000	0.0001
Cutibacterium_acnes	0.0000	0.0000	0.0000	0.0000	0.0000	0.0000
*Actinobaculum*_sp_oral_taxon_183	0.0000	0.0020	0.0000	0.0000	0.0000	0.0003
Actinomyces_graevenitzii	0.0309	0.0361	0.0078	0.0321	0.0311	0.0558
*Actinomyces*_sp_oral_taxon_897	0.0000	0.0004	0.0000	0.0002	0.0000	0.0001
Actinomyces_johnsonii	0.0000	0.0001	0.0000	0.0000	0.0000	0.0004
Actinomyces_viscosus	0.0000	0.0003	0.0000	0.0000	0.0000	0.0004
Actinomyces_naeslundii	0.0023	0.0085	0.0000	0.0081	0.0037	0.0332
*Actinomyces*_sp_oral_taxon_170	0.0000	0.0000	0.0000	0.0000	0.0000	0.0000
Actinomyces_oris	0.0016	0.0067	0.0000	0.0009	0.0008	0.0041
*Actinomyces*_sp_oral_taxon_414	0.0000	0.0003	0.0000	0.0006	0.0000	0.0000
Actinomyces_massiliensis	0.0000	0.0010	0.0000	0.0000	0.0000	0.0002
*Actinomyces*_sp_oral_taxon_448	0.0000	0.0024	0.0000	0.0000	0.0000	0.0000
Actinomyces_turicensis	0.0000	0.0000	0.0000	0.0000	0.0000	0.0000
Actinomyces_cardiffensis	0.0000	0.0000	0.0000	0.0000	0.0000	0.0001
*Actinomyces*_sp_oral_taxon_181	0.0022	0.0120	0.0000	0.0162	0.0020	0.0130
*Actinomyces*_sp_S6_Spd3	0.0012	0.0040	0.0016	0.0038	0.0027	0.0052
Actinomyces_georgiae	0.0000	0.0000	0.0000	0.0000	0.0000	0.0000
Actinomyces_hongkongensis	0.0000	0.0000	0.0000	0.0000	0.0000	0.0001
Actinomyces_meyeri	0.0000	0.0000	0.0000	0.0003	0.0000	0.0000
*Actinomyces*_sp_ICM47	0.0314	0.0328	0.0002	0.0180	0.0277	0.0591
Actinomyces_odontolyticus	0.0085	0.0059	0.0077	0.0080	0.0108	0.0146
*Actinomyces*_sp_HMSC035G02	0.0052	0.0104	0.0011	0.0108	0.0062	0.0169
*Actinomyces*_sp_oral_taxon_180	0.0000	0.0003	0.0000	0.0021	0.0000	0.0026
*Actinomyces*_sp_HPA0247	0.0007	0.0055	0.0001	0.0600	0.0004	0.0067
Alloscardovia_omnicolens	0.0000	0.0066	0.0000	0.0003	0.0000	0.0003

Parascardovia_denticolens	0.0000	0.0000	0.0000	0.0002	0.0000	0.0001
Scardovia_wiggsiae	0.0000	0.0084	0.0000	0.0001	0.0000	0.0006
Gardnerella_vaginalis	0.0000	0.0000	0.0000	0.0000	0.0000	0.0000
Bifidobacterium_scardovii	0.0000	0.0000	0.0000	0.0000	0.0000	0.0000
Bifidobacterium_dentium	0.0000	0.0000	0.0000	0.0001	0.0000	0.0025
Bifidobacterium_longum	0.0000	0.0000	0.0000	0.0018	0.0000	0.0064
Micrococcus_luteus	0.0000	0.0000	0.0000	0.0000	0.0000	0.0000
Rothia_aeria	0.0022	0.1240	0.0000	0.0136	0.0056	0.0213
Rothia_mucilaginosa	0.1235	0.0724	0.0675	0.1383	0.0870	0.0504
Rothia_dentocariosa	0.0032	0.0326	0.0000	0.0099	0.0097	0.0561
Slackia_exigua	0.0000	0.0000	0.0000	0.0001	0.0000	0.0000
Atopobium_minutum	0.0000	0.0000	0.0000	0.0000	0.0000	0.0000
Atopobium_parvulum	0.0003	0.0014	0.0010	0.0043	0.0015	0.0022
Atopobium_rimae	0.0000	0.0012	0.0002	0.0029	0.0007	0.0026
Olsenella_uli	0.0000	0.0000	0.0000	0.0004	0.0000	0.0000
Olsenella_scatoligenes	0.0000	0.0000	0.0000	0.0000	0.0000	0.0000
Dialister_micraerophilus	0.0000	0.0000	0.0000	0.0002	0.0000	0.0000
Dialister_pneumosintes	0.0000	0.0005	0.0000	0.0003	0.0000	0.0004
Dialister_invisus	0.0000	0.0000	0.0000	0.0000	0.0000	0.0000
Megasphaera_micronuciformis	0.0024	0.0159	0.0035	0.0129	0.0046	0.0132
Anaeroglobus_geminatus	0.0000	0.0000	0.0000	0.0001	0.0000	0.0000
Veillonella_atypica	0.0304	0.0514	0.0124	0.0370	0.0300	0.0394
Veillonella_tobetsuensis	0.0000	0.0019	0.0000	0.0014	0.0000	0.0037
Veillonella_parvula	0.0037	0.0078	0.0019	0.0072	0.0021	0.0101
Veillonella_rodentium	0.0000	0.0000	0.0000	0.0000	0.0000	0.0000
*Veillonella*_sp_T11011_6	0.0010	0.0020	0.0013	0.0022	0.0007	0.0019
Veillonella_infantium	0.0046	0.0026	0.0043	0.0059	0.0035	0.0048
Veillonella_dispar	0.0171	0.0135	0.0131	0.0309	0.0095	0.0179
Veillonella_rogosae	0.0000	0.0022	0.0000	0.0007	0.0000	0.0013
*Mitsuokella*_sp_oral_taxon_131	0.0000	0.0000	0.0000	0.0000	0.0000	0.0000
Selenomonas_sputigena	0.0000	0.0004	0.0000	0.0000	0.0000	0.0009
Selenomonas_infelix	0.0000	0.0000	0.0000	0.0000	0.0000	0.0000
*Selenomonas*_sp_oral_taxon_920	0.0000	0.0000	0.0000	0.0000	0.0000	0.0003
*Selenomonas*_sp_FOBRC6	0.0000	0.0000	0.0000	0.0000	0.0000	0.0000
*Selenomonas*_sp_oral_taxon_138	0.0000	0.0000	0.0000	0.0000	0.0000	0.0000
*Selenomonas*_sp_oral_taxon_892	0.0000	0.0000	0.0000	0.0000	0.0000	0.0003
Selenomonas_noxia	0.0000	0.0000	0.0000	0.0000	0.0000	0.0000
Selenomonas_artemidis	0.0000	0.0000	0.0000	0.0000	0.0000	0.0002
Selenomonas_flueggei	0.0000	0.0003	0.0000	0.0000	0.0000	0.0003
Centipeda_periodontii	0.0000	0.0000	0.0000	0.0000	0.0000	0.0000
*Selenomonas*_sp_F0473	0.0000	0.0000	0.0000	0.0000	0.0000	0.0000
*Selenomonas*_sp_oral_taxon_126	0.0000	0.0000	0.0000	0.0000	0.0000	0.0000
Pseudoramibacter_alactolyticus	0.0000	0.0000	0.0000	0.0000	0.0000	0.0000
*Peptostreptococcaceae*_bacterium_oral_taxon_113	0.0000	0.0000	0.0000	0.0000	0.0000	0.0000
*Parvimonas*_sp_oral_taxon_110	0.0000	0.0000	0.0000	0.0000	0.0000	0.0000

*Parvimonas*_sp_oral_taxon_393	0.0000	0.0000	0.0000	0.0000	0.0000	0.0000
Parvimonas_micra	0.0000	0.0014	0.0001	0.0059	0.0000	0.0023
Filifactor_alocis	0.0000	0.0007	0.0000	0.0001	0.0000	0.0004
Peptoanaerobacter_stomatis	0.0000	0.0000	0.0000	0.0000	0.0000	0.0000
Eubacterium_yurii	0.0000	0.0000	0.0000	0.0000	0.0000	0.0001
Peptostreptococcus_stomatis	0.0000	0.0001	0.0000	0.0004	0.0000	0.0004
Eubacterium_saphenum	0.0000	0.0001	0.0000	0.0001	0.0000	0.0001
Eubacterium_brachy	0.0000	0.0040	0.0005	0.0057	0.0002	0.0028
Mogibacterium_timidum	0.0000	0.0013	0.0000	0.0005	0.0000	0.0012
Mogibacterium_pumilum	0.0000	0.0000	0.0000	0.0000	0.0000	0.0000
Mogibacterium_diversum	0.0006	0.0038	0.0000	0.0039	0.0022	0.0044
Eubacterium_sulci	0.0006	0.0027	0.0000	0.0042	0.0014	0.0043
Eubacterium_infirmum	0.0000	0.0001	0.0000	0.0005	0.0000	0.0000
Eubacterium_nodatum	0.0000	0.0024	0.0000	0.0012	0.0000	0.0010
Eubacterium_minutum	0.0000	0.0000	0.0000	0.0000	0.0000	0.0000
Catonella_morbi	0.0000	0.0000	0.0000	0.0000	0.0000	0.0001
*Lachnospiraceae*_bacterium_oral_taxon_096	0.0000	0.0020	0.0000	0.0010	0.0000	0.0013
Johnsonella_ignava	0.0000	0.0000	0.0000	0.0000	0.0000	0.0000
Lachnoanaerobaculum_saburreum	0.0000	0.0013	0.0000	0.0018	0.0005	0.0015
Lachnoanaerobaculum_umeaense	0.0000	0.0005	0.0000	0.0000	0.0000	0.0013
Stomatobaculum_longum	0.0003	0.0024	0.0015	0.0044	0.0009	0.0031
*Oribacterium*_sp_oral_taxon_078	0.0000	0.0000	0.0000	0.0001	0.0000	0.0000
Oribacterium_sinus	0.0005	0.0050	0.0001	0.0008	0.0005	0.0012
Oribacterium_asaccharolyticum	0.0000	0.0000	0.0000	0.0001	0.0000	0.0000
Oribacterium_parvum	0.0000	0.0008	0.0000	0.0009	0.0001	0.0021
Shuttleworthia_satelles	0.0000	0.0000	0.0000	0.0002	0.0000	0.0000
Brochothrix_thermosphacta	0.0000	0.0000	0.0000	0.0065	0.0000	0.0000
Eggerthia_catenaformis	0.0000	0.0005	0.0000	0.0001	0.0000	0.0008
Solobacterium_moorei	0.0000	0.0002	0.0000	0.0007	0.0002	0.0004
Gemella_morbillorum	0.0003	0.0012	0.0002	0.0009	0.0009	0.0033
Gemella_sanguinis	0.0254	0.0282	0.0221	0.0258	0.0391	0.0294
Gemella_haemolysans	0.0015	0.0041	0.0000	0.0089	0.0019	0.0153
Abiotrophia_defectiva	0.0000	0.0000	0.0000	0.0001	0.0000	0.0003
*Abiotrophia*_sp_HMSC24B09	0.0000	0.0008	0.0000	0.0008	0.0000	0.0029
Granulicatella_adiacens	0.0000	0.0003	0.0000	0.0205	0.0001	0.0104
Enterococcus_faecalis	0.0000	0.0000	0.0000	0.0099	0.0000	0.0000
Lactococcus_lactis	0.0000	0.0007	0.0000	0.0000	0.0000	0.0000
Streptococcus_peroris	0.0000	0.0004	0.0000	0.0036	0.0000	0.0012
Streptococcus_infantis	0.0294	0.0401	0.0506	0.0731	0.0350	0.0261
Streptococcus_cristatus	0.0001	0.0017	0.0000	0.0004	0.0000	0.0008
Streptococcus_sanguinis	0.0079	0.0225	0.0000	0.0132	0.0101	0.0198
*Streptococcus*_sp_oral_taxon_056	0.0000	0.0000	0.0000	0.0000	0.0000	0.0007
*Streptococcus*_sp_F0442	0.0003	0.0024	0.0000	0.0003	0.0000	0.0010
*Streptococcus*_sp_A12	0.0000	0.0018	0.0000	0.0023	0.0000	0.0022
Streptococcus_australis	0.0030	0.0059	0.0000	0.0115	0.0031	0.0053

Streptococcus_gordonii	0.0000	0.0048	0.0000	0.0206	0.0000	0.0031
Streptococcus_milleri	0.0000	0.0005	0.0000	0.0018	0.0000	0.0002
Streptococcus_anginosus_group	0.0000	0.0003	0.0000	0.0015	0.0000	0.0018
Streptococcus_viridans	0.0000	0.0000	0.0000	0.0003	0.0000	0.0014
Streptococcus_parasanguinis	0.0490	0.0586	0.0747	0.1413	0.0457	0.0742
*Streptococcus*_sp_HMSC034E03	0.0005	0.0021	0.0007	0.0025	0.0006	0.0022
*Streptococcus*_sp_HMSC071D03	0.0000	0.0004	0.0000	0.0003	0.0000	0.0002
*Streptococcus*_sp_HMSC067H01	0.0003	0.0022	0.0007	0.0023	0.0002	0.0017
*Streptococcus*_sp_HPH0090	0.0000	0.0002	0.0000	0.0000	0.0000	0.0002
Streptococcus_mitis	0.0121	0.0227	0.0015	0.0545	0.0252	0.0646
*Streptococcus*_sp_M334	0.0000	0.0003	0.0000	0.0001	0.0000	0.0006
Streptococcus_pseudopneumoniae	0.0000	0.0022	0.0000	0.0011	0.0000	0.0022
*Streptococcus*_sp_HMSC070B10	0.0000	0.0000	0.0000	0.0046	0.0000	0.0000
*Streptococcus*_sp_oral_taxon_058	0.0000	0.0000	0.0000	0.0003	0.0000	0.0000
Streptococcus_oralis	0.0103	0.0091	0.0131	0.0673	0.0132	0.0144
*Streptococcus*_sp_NLAE_zl_C503	0.0000	0.0001	0.0000	0.0000	0.0000	0.0000
Streptococcus_mutans	0.0000	0.0004	0.0000	0.0000	0.0000	0.0000
Streptococcus_sobrinus	0.0000	0.0000	0.0000	0.0000	0.0000	0.0000
Streptococcus_thermophilus	0.0000	0.0007	0.0000	0.0000	0.0000	0.0000
Streptococcus_vestibularis	0.0000	0.0009	0.0000	0.0001	0.0000	0.0008
*Streptococcus_salivarius*_CAG_79	0.0000	0.0001	0.0000	0.0000	0.0000	0.0000
Streptococcus_salivarius	0.0625	0.0669	0.0087	0.0202	0.0271	0.0416
Leuconostoc_carnosum	0.0000	0.0000	0.0000	0.0004	0.0000	0.0000
Lactobacillus_rhamnosus	0.0000	0.0000	0.0000	0.0011	0.0000	0.0000
Lactobacillus_kalixensis	0.0000	0.0148	0.0000	0.0000	0.0000	0.0078
Lactobacillus_gasseri	0.0000	0.0000	0.0000	0.0001	0.0000	0.0005
Lactobacillus_salivarius	0.0000	0.0000	0.0000	0.0001	0.0000	0.0028
Lactobacillus_fermentum	0.0000	0.0061	0.0000	0.0476	0.0000	0.0058
Lactobacillus_vaginalis	0.0000	0.0014	0.0000	0.0000	0.0000	0.0000
*Bacteroidetes*_oral_taxon_274	0.0000	0.0000	0.0000	0.0006	0.0000	0.0002
Tannerella_forsythia	0.0000	0.0027	0.0000	0.0003	0.0000	0.0022
*Tannerella*_sp_oral_taxon_HOT_286	0.0000	0.0026	0.0000	0.0026	0.0002	0.0041
*Tannerella*_sp_oral_taxon_808	0.0000	0.0001	0.0000	0.0002	0.0000	0.0000
Porphyromonas_canoris	0.0000	0.0000	0.0000	0.0000	0.0000	0.0000
Porphyromonas_endodontalis	0.0000	0.0016	0.0001	0.0019	0.0000	0.0015
Porphyromonas_gingivalis	0.0000	0.0142	0.0000	0.0156	0.0000	0.0081
*Porphyromonas*_sp_oral_taxon_278	0.0000	0.0000	0.0000	0.0000	0.0000	0.0000
Porphyromonas_catoniae	0.0000	0.0002	0.0000	0.0000	0.0000	0.0006
Porphyromonas_somerae	0.0002	0.0118	0.0016	0.0209	0.0061	0.0178
*Bacteroidetes*_bacterium_oral_taxon_272	0.0000	0.0000	0.0000	0.0000	0.0000	0.0000
Bacteroides_heparinolyticus	0.0000	0.0000	0.0000	0.0000	0.0000	0.0000
*Prevotella*_sp_oral_taxon_473	0.0000	0.0002	0.0000	0.0001	0.0000	0.0007
Alloprevotella_tannerae	0.0010	0.0063	0.0014	0.0108	0.0048	0.0071
Alloprevotella_rava	0.0000	0.0013	0.0000	0.0002	0.0000	0.0009
Prevotella_pleuritidis	0.0000	0.0017	0.0000	0.0012	0.0000	0.0004

Prevotella_enoeca	0.0000	0.0000	0.0000	0.0000	0.0000	0.0000
Prevotella_marshii	0.0000	0.0000	0.0000	0.0000	0.0000	0.0000
*Prevotella*_sp_oral_taxon_299	0.0000	0.0001	0.0001	0.0005	0.0000	0.0002
Prevotella_saccharolytica	0.0000	0.0000	0.0000	0.0000	0.0000	0.0000
Prevotella_loescheii	0.0000	0.0003	0.0000	0.0000	0.0000	0.0003
Prevotella_shahii	0.0000	0.0031	0.0000	0.0008	0.0000	0.0044
Prevotella_micans	0.0000	0.0000	0.0000	0.0000	0.0000	0.0000
Prevotella_multiformis	0.0000	0.0002	0.0000	0.0000	0.0000	0.0001
Prevotella_histicola	0.0033	0.0402	0.0000	0.0105	0.0024	0.0266
Prevotella_denticola	0.0000	0.0017	0.0000	0.0011	0.0000	0.0013
Prevotella_veroralis	0.0000	0.0001	0.0000	0.0001	0.0000	0.0000
*Prevotella*_sp_oral_taxon_306	0.0017	0.0165	0.0000	0.0193	0.0004	0.0074
Prevotella_fusca	0.0000	0.0000	0.0000	0.0000	0.0000	0.0000
Prevotella_scopos	0.0000	0.0009	0.0000	0.0004	0.0000	0.0008
*Prevotella*_sp_F0091	0.0000	0.0000	0.0000	0.0000	0.0000	0.0000
Prevotella_melaninogenica	0.0187	0.0384	0.0061	0.0400	0.0204	0.0371
Prevotella_jejuni	0.0003	0.0280	0.0000	0.0072	0.0055	0.0162
Prevotella_intermedia	0.0000	0.0151	0.0000	0.0094	0.0000	0.0137
Prevotella_nigrescens	0.0006	0.0142	0.0001	0.0021	0.0015	0.0034
Prevotella_pallens	0.0061	0.0263	0.0022	0.0213	0.0105	0.0232
Prevotella_oulorum	0.0000	0.0019	0.0000	0.0003	0.0002	0.0087
Prevotella_baroniae	0.0000	0.0018	0.0000	0.0003	0.0000	0.0000
Prevotella_dentalis	0.0000	0.0010	0.0000	0.0002	0.0000	0.0007
Prevotella_buccae	0.0000	0.0027	0.0000	0.0000	0.0000	0.0001
Prevotella_oralis	0.0000	0.0000	0.0000	0.0000	0.0000	0.0000
*Prevotella*_sp_oral_taxon_376	0.0000	0.0000	0.0000	0.0000	0.0000	0.0000
Prevotella_oris	0.0001	0.0010	0.0001	0.0010	0.0002	0.0009
Prevotella_maculosa	0.0000	0.0000	0.0000	0.0000	0.0000	0.0000
Prevotella_salivae	0.0012	0.0102	0.0000	0.0095	0.0036	0.0048
Capnocytophaga_haemolytica	0.0000	0.0000	0.0000	0.0000	0.0000	0.0000
Capnocytophaga_ochracea	0.0000	0.0002	0.0000	0.0001	0.0000	0.0000
Capnocytophaga_sputigena	0.0000	0.0052	0.0011	0.0508	0.0003	0.0019
Capnocytophaga_leadbetteri	0.0000	0.0042	0.0022	0.0104	0.0011	0.0039
*Capnocytophaga*_sp_oral_taxon_332	0.0000	0.0001	0.0000	0.0000	0.0000	0.0001
Capnocytophaga_granulosa	0.0000	0.0002	0.0000	0.0012	0.0000	0.0004
*Capnocytophaga*_sp_oral_taxon_338	0.0000	0.0000	0.0000	0.0000	0.0000	0.0000
*Capnocytophaga*_sp_oral_taxon_863	0.0000	0.0001	0.0000	0.0000	0.0000	0.0001
Capnocytophaga_gingivalis	0.0000	0.0053	0.0000	0.0008	0.0007	0.0021
Desulfobulbus_oralis	0.0000	0.0003	0.0000	0.0000	0.0000	0.0001
Bilophila_wadsworthia	0.0000	0.0000	0.0000	0.0000	0.0000	0.0000
Neisseria_bacilliformis	0.0000	0.0007	0.0000	0.0028	0.0000	0.0027
Neisseria_elongata	0.0000	0.0081	0.0000	0.0013	0.0000	0.0152
Eikenella_corrodens	0.0000	0.0010	0.0000	0.0013	0.0000	0.0006
*Eikenella*_sp_NML130454	0.0000	0.0001	0.0000	0.0009	0.0000	0.0002
Kingella_denitrificans	0.0000	0.0005	0.0000	0.0000	0.0000	0.0001
Kingella_oralis	0.0000	0.0015	0.0000	0.0011	0.0000	0.0008

Simonsiella_muelleri	0.0000	0.0000	0.0000	0.0000	0.0000	0.0000
Neisseria_subflava	0.0000	0.0054	0.0000	0.0128	0.0001	0.0046
Neisseria_flavescens	0.0259	0.0966	0.0318	0.1528	0.0721	0.0860
Neisseria_perflava	0.0000	0.0011	0.0000	0.0000	0.0000	0.0020
Neisseria_cinerea	0.0000	0.0000	0.0000	0.0000	0.0000	0.0013
Morococcus_cerebrosus	0.0000	0.0001	0.0000	0.0000	0.0000	0.0019
Neisseria_sicca	0.0007	0.0104	0.0000	0.1280	0.0006	0.0078
*Neisseria*_sp_oral_taxon_014	0.0000	0.0175	0.0000	0.0006	0.0000	0.0036
*Neisseria*_sp_HMSC064E01	0.0000	0.0000	0.0000	0.0002	0.0000	0.0000
Neisseria_macacae	0.0000	0.0010	0.0000	0.0013	0.0000	0.0001
Neisseria_mucosa	0.0000	0.0007	0.0000	0.0014	0.0000	0.0013
Lautropia_mirabilis	0.0016	0.0163	0.0003	0.0055	0.0024	0.0136
*Ottowia*_sp_oral_taxon_894	0.0000	0.0010	0.0000	0.0005	0.0000	0.0001
Cardiobacterium_hominis	0.0000	0.0021	0.0000	0.0004	0.0000	0.0017
Cardiobacterium_valvarum	0.0000	0.0018	0.0000	0.0000	0.0000	0.0003
Haemophilus_parahaemolyticus	0.0000	0.0027	0.0000	0.0000	0.0000	0.0030
Haemophilus_paraphrohaemolyticus	0.0000	0.0003	0.0000	0.0000	0.0000	0.0003
Haemophilus_sputorum	0.0000	0.0083	0.0000	0.0137	0.0000	0.0088
Aggregatibacter_aphrophilus	0.0000	0.0015	0.0000	0.0048	0.0000	0.0005
Aggregatibacter_actinomycetemcomitans	0.0000	0.0000	0.0000	0.0000	0.0000	0.0004
*Aggregatibacter*_sp_oral_taxon_458	0.0000	0.0006	0.0000	0.0000	0.0000	0.0007
Aggregatibacter_segnis	0.0000	0.0016	0.0000	0.0000	0.0000	0.0011
Haemophilus_haemolyticus	0.0000	0.0005	0.0000	0.0006	0.0000	0.0004
Haemophilus_quentini	0.0000	0.0000	0.0000	0.0000	0.0000	0.0000
*Haemophilus*_sp_HMSC71H05	0.0004	0.0027	0.0000	0.0122	0.0000	0.0024
Haemophilus_parainfluenzae	0.0129	0.0186	0.0236	0.1019	0.0179	0.0160
Haemophilus_pittmaniae	0.0000	0.0002	0.0000	0.0018	0.0000	0.0011

Supplementary Tables

Supplementary-Table 3: Enrichment of ARGs in plaque and saliva T2 vs. T1.

	Gene	PRODUCT	RESISTANCE	Estimate	p-value	FDR
Plaque T2 vs. T1	*tetB*(60)	tetracycline efflux ABC transporter Tet(60) subunit B	TETRACYCLINE	2.3536	0.0026	0.0546
	erm(X)	23S rRNA (adenine(2058)-N(6))-methyltransferase Erm(X)	MACROLIDE	-2.3254	0.0392	0.3650
	lsa(C)	ABC-F type ribosomal protection protein Lsa(C)	LINCOSAMIDE;STREPTOGRAMIN	-1.0986	0.1078	0.7160
	tetA(60)	tetracycline efflux ABC transporter Tet(60) subunit A	TETRACYCLINE	1.3499	0.1297	0.8041
	cfxA3	class A extended-spectrum beta-lactamase CfxA3	BETA-LACTAM	1.5581	0.1823	0.9138
	tet(32)	tetracycline resistance ribosomal protection protein Tet(32)	TETRACYCLINE	-0.6931	0.3145	1.0000
	erm(B)	23S rRNA (adenine(2058)-N(6))-methyltransferase Erm(B)	MACROLIDE	0.6466	0.3297	1.0000
	tet(M)	tetracycline resistance ribosomal protection protein Tet(M)	TETRACYCLINE	0.6190	0.3394	1.0000
	tet(Q)	tetracycline resistance ribosomal protection protein Tet(Q)	TETRACYCLINE	-0.6061	0.3440	1.0000
	cfxA_gen	CfxA family class A broad-spectrum beta-lactamase	BETA-LACTAM	-0.4013	0.5278	1.0000
	cfxA	class A broad-spectrum beta-lactamase CfxA	BETA-LACTAM	-0.7472	0.5557	1.0000
	catP	type A-11 chloramphenicol O-acetyltransferase CatP	CHLORAMPHENICOL	-0.7472	0.5557	1.0000
	mef(A)	macrolide efflux MFS transporter Mef(A)	MACROLIDE	0.2513	0.7235	1.0000
	erm(F)	23S rRNA (adenine(2058)-N(6))-methyltransferase Erm(F)	MACROLIDE	0.2136	0.7441	1.0000
	tet(W)	tetracycline resistance ribosomal protection protein Tet(W)	TETRACYCLINE	-18.4675	0.9939	1.0000
	blaCSP-1	class A extended-spectrum beta-lactamase CSP-1	CEPHALOSPORIN	17.8315	0.9941	1.0000
	aph(3')-IIIa	aminoglycoside O-phosphotransferase APH(3')-IIIa	AMIKACIN;KANAMYCIN	17.8315	0.9941	1.0000
	tet(B)	tetracycline efflux MFS transporter Tet(B)	TETRACYCLINE	19.1798	0.9961	1.0000
	lnu(AN2)	lincosamide nucleotidyltransferase Lnu(AN2)	LINCOSAMIDE	-17.6216	0.9965	1.0000
	*sat*4	streptothricin N-acetyltransferase Sat4	STREPTOTHRICIN	19.3688	0.9976	1.0000
	aph(3'')-Ib	aminoglycoside O-phosphotransferase APH(3'')-Ib	STREPTOMYCIN	18.6216	0.9977	1.0000
	aph(3')-Ia	aminoglycoside O-phosphotransferase APH(3')-Ia	KANAMYCIN	18.6216	0.9977	1.0000
	aph(6)-Id	aminoglycoside O-phosphotransferase APH(6)-Id	STREPTOMYCIN	18.6216	0.9977	1.0000
	tet(A)	tetracycline efflux MFS transporter Tet(A)	TETRACYCLINE	18.6216	0.9977	1.0000
	dfrE	trimethoprim-resistant dihydrofolate reductase DfrE	TRIMETHOPRIM	18.6216	0.9977	1.0000
	lsa(A)	ABC-F type ribosomal protection protein Lsa(A)	LINCOSAMIDE;STREPTOGRAMIN	18.6216	0.9977	1.0000
	tetA(46)	tetracycline efflux ABC transporter Tet(46) subunit A	TETRACYCLINE	18.6216	0.9977	1.0000
	tetB(46)	tetracycline efflux ABC transporter Tet(46) subunit B	TETRACYCLINE	18.6216	0.9977	1.0000
	nimI	nitroimidazole resistance protein NimI	NITROIMIDAZOLE	-18.6216	0.9977	1.0000
	msr(D)	ABC-F type ribosomal protection protein Msr(D)	MACROLIDE	0.0000	1.0000	1.0000
	tet(O)	tetracycline resistance ribosomal protection protein Tet(O)	TETRACYCLINE	0.0000	1.0000	1.0000
	Gene	PRODUCT	RESISTANCE	Estimate	p-value	FDR
Saliva T2 vs. T1	*tet*(B)	tetracycline efflux MFS transporter Tet(B)	TETRACYCLINE	3.0845	0.0071	0.0734
	tetB(60)	tetracycline efflux ABC transporter Tet(60) subunit B	TETRACYCLINE	2.0448	0.0784	0.4289
	tetB(46)	tetracycline efflux ABC transporter Tet(46) subunit B	TETRACYCLINE	1.2528	0.0975	0.4780
	tetA(46)	tetracycline efflux ABC transporter Tet(46) subunit A	TETRACYCLINE	1.2068	0.0977	0.4780
	erm(X)	23S rRNA (adenine(2058)-N(6))-methyltransferase Erm(X)	MACROLIDE	1.2910	0.1623	0.7545
	tetA(60)	tetracycline efflux ABC transporter Tet(60) subunit A	TETRACYCLINE	1.3669	0.2594	1.0000
	tet(W)	tetracycline resistance ribosomal protection protein Tet(W)	TETRACYCLINE	-0.5653	0.4313	1.0000
	sat4	streptothricin N-acetyltransferase Sat4	STREPTOTHRICIN	0.8873	0.4871	1.0000
	erm(B)	23S rRNA (adenine(2058)-N(6))-methyltransferase Erm(B)	MACROLIDE	0.5108	0.5508	1.0000
	erm(F)	23S rRNA (adenine(2058)-N(6))-methyltransferase Erm(F)	MACROLIDE	0.5108	0.5508	1.0000
	tet(M)	tetracycline resistance ribosomal protection protein Tet(M)	TETRACYCLINE	0.4643	0.5529	1.0000
	cfxA_gen	CfxA family class A broad-spectrum beta-lactamase	BETA-LACTAM	-0.3365	0.6423	1.0000
	cfxA3	class A extended-spectrum beta-lactamase CfxA3	BETA-LACTAM	-0.2136	0.8028	1.0000
	mef(A)	macrolide efflux MFS transporter Mef(A)	MACROLIDE	-0.1335	0.9002	1.0000
	msr(D)	ABC-F type ribosomal protection protein Msr(D)	MACROLIDE	-0.1335	0.9002	1.0000
	tet(Q)	tetracycline resistance ribosomal protection protein Tet(Q)	TETRACYCLINE	-0.0282	0.9675	1.0000
	lsa(C)	ABC-F type ribosomal protection protein Lsa(C)	LINCOSAMIDE;STREPTOGRAMIN	18.0997	0.9943	1.0000
	aph(3')-IIIa	aminoglycoside O-phosphotransferase APH(3')-IIIa	AMIKACIN;KANAMYCIN	17.6202	0.9945	1.0000
	tet(O)	tetracycline resistance ribosomal protection protein Tet(O)	TETRACYCLINE	19.0997	0.9964	1.0000
	tet(32)	tetracycline resistance ribosomal protection protein Tet(32)	TETRACYCLINE	-17.7329	0.9968	1.0000
	aph(3'')-Ib	aminoglycoside O-phosphotransferase APH(3'')-Ib	STREPTOMYCIN	0.0000	1.0000	1.0000
	aph(3')-Ia	aminoglycoside O-phosphotransferase APH(3')-Ia	KANAMYCIN	0.0000	1.0000	1.0000
	aph(6)-Id	aminoglycoside O-phosphotransferase APH(6)-Id	STREPTOMYCIN	0.0000	1.0000	1.0000
	blaCSP-1	class A extended-spectrum beta-lactamase CSP-1	CEPHALOSPORIN	0.0000	1.0000	1.0000

	catP	type A-11 chloramphenicol O-acetyltransferase CatP	CHLORAMPHENICOL	0.0000	1.0000	1.0000
	cfxA	class A broad-spectrum beta-lactamase CfxA	BETA-LACTAM	0.0000	1.0000	1.0000
	dfrE	trimethoprim-resistant dihydrofolate reductase DfrE	TRIMETHOPRIM	0.0000	1.0000	1.0000
	lnu(AN2)	lincosamide nucleotidyltransferase Lnu(AN2)	LINCOSAMIDE	0.0000	1.0000	1.0000
	lsa(A)	ABC-F type ribosomal protection protein Lsa(A)	LINCOSAMIDE;STREPTOGRAMIN	0.0000	1.0000	1.0000
	nimI	nitroimidazole resistance protein NimI	NITROIMIDAZOLE	0.0000	1.0000	1.0000
	tet(A)	tetracycline efflux MFS transporter Tet(A)	TETRACYCLINE	0.0000	1.0000	1.0000
